# The scion-driven transcriptomic changes guide the resilience of grafted near-isohydric grapevines under water deficit

**DOI:** 10.1093/hr/uhae291

**Published:** 2024-10-23

**Authors:** Alberto Rodriguez-Izquierdo, David Carrasco, Luis Valledor, Josefina Bota, Cristina López-Hidalgo, Maria A Revilla, Rosa Arroyo-Garcia

**Affiliations:** Centro de Biotecnología y Genómica de Plantas (CBGP-INIA), CSIC – Universidad Politécnica de Madrid, Campus Montegancedo, Madrid, Spain; Centro de Biotecnología y Genómica de Plantas (CBGP-INIA), CSIC – Universidad Politécnica de Madrid, Campus Montegancedo, Madrid, Spain; Department of Organisms and Systems Biology, Institute of Biotechnology of Asturias, University of Oviedo, 33071 Oviedo, Spain; Research Group on Plant Biology under Mediterranean Conditions (PlantMed), Biology Department, Agro-Environmental and Water Economy Institute-Universitat de les Illes Balears (INAGEA), Universitat de les Illes Balears (UIB), Carretera de Valldemossa, km 7.5, 07122 Palma, Spain; Department of Organisms and Systems Biology, Institute of Biotechnology of Asturias, University of Oviedo, 33071 Oviedo, Spain; Department of Organisms and Systems Biology, Institute of Biotechnology of Asturias, University of Oviedo, 33071 Oviedo, Spain; Centro de Biotecnología y Genómica de Plantas (CBGP-INIA), CSIC – Universidad Politécnica de Madrid, Campus Montegancedo, Madrid, Spain

## Abstract

The large diversity of grapevine cultivars includes genotypes more tolerant to water deficit than others. Widely distributed cultivars, like *Merlot,* are more sensitive to water deprivation than local cultivars like *Callet*, which are more adapted to water deficit due to their Mediterranean origin. Despite their tolerance, adaptation to water deficit influenced by grafting in rootstocks like *110 Richter* is key to facing drought in vineyards, defining the scion–rootstock relationship. To understand these differences, we explored transcriptomic, metabolic, hormonal and physiological responses under three levels of water deficit (mild, high, and extreme), using *110 Richter* as the rootstock in both cultivars. Results revealed that sensitivity to abscisic acid (ABA) is essential for water deficit tolerance in the aerial part, guiding root responses. *Callet/110 Richter* activates more gene expression patterns in response to ABA, reducing water loss compared to *Merlot/110 Richter* in both aerial and root parts. This modulation in *Callet/110 Richter* involves regulating metabolic pathways to increase cell turgor, reducing photosynthesis, and producing molecules like polyphenols or flavonoids to respond to oxidative stress. In contrast, Merlot/110 Richter shows a lack of specific response, especially in the roots, indicating less resilience to water stress. Therefore, selecting genotypes more sensitive to ABA and their interaction with rootstocks is key for managing vineyards in future climate change scenarios.

## Introduction

The grapevine (*Vitis vinifera* L.) is one of the most cultivated plants in the world. Due to the large surface of the crop (e.g. in Spain, the vineyard extension reaches 950.000 ha) [1], the grapevine has a substantial economic impact, generating ~1.000–1.500 million euros annually, representing ~10% of the total agriculture production value [1]. It also plays a crucial role in shaping landscapes, providing ecosystem services, and offering a variety of products [1, 2]. However, the current climate change scenario is affecting grapevine production, mainly through prolonged and intensified periods of water deficit, which reduces both yield and berry quality [3]. Nonetheless, not all grapevine genotypes react in the same way to these conditions, offering a possible solution to reduce the damages in vineyards by the study of alternative genotypes coming from local cultivars commonly grown at regions with low water availability [4–6].

The different responses to water deficit in grapevines can be categorized as either near-isohydric (‘pessimistic’) or near-anisohydric (‘optimistic’), primarily determined by stomatal behavior and physiological adaptations to water status. Near-isohydric behavior involves conserving current resources and controlling demand for future events, while anisohydric behavior uses available resources with the expectation of future replenishment [7]. Although this classification is somewhat imprecise [4], it provides a basic differentiation in grapevine genotypic diversity. Parameters like stomatal conductance, hydric stomatal pressure, vapor pressure deficit, or soil water potential can help establish varying levels of water use efficiency (WUE) based on physiological changes in grapevines [4, 8–10]. For example, the globally popular *Merlot* cultivar is considered an anisohydric genotype [11], as it exhibits less stomatal closure regulation, varying them the WUE respect from other cultivars. In contrast, the local Balearic Islands cultivar *Callet*, known for its interesting wine profile [12], is considered near-isohydric due to better control of stomatal closure and water optimization [4–6]. Water deficit stress triggers common responses in plants, including morphological and physiological changes to minimize damage. These responses often involve reduced aerial growth, lower evapotranspiration to limit water loss, and decreased photosynthesis rates, which may be compromised by oxidative stress and generation of Reactive Oxygen Species (ROS) under water deficit conditions [13, 14]. However, not all grapevine genotypes respond uniformly to water deficit stress, highlighting the importance of studying physiological variation within the grapevine genetic pool to enhance resilience to climate change.

The physiological and morphological changes driven by water deprivation in grapevines are closely linked to specific metabolic pathways and phytohormone production. Water deficit alters hormonal rates in response to stress, involving changes in abscisic acid (ABA), jasmonic acid (Jas), auxins, or gibberellins (Gas), with ABA being one of the earliest to abiotic stress in grapevine plants [15, 16]. These hormonal changes regulate key processes such as the regulation of sucrose transport to the phloem and the regulation of polyphenol and osmolyte biosynthesis [17]. By examining these responses, we can better understand variations in metabolite and hormone reactions, which are crucial for identifying genotypes better suited to withstand climate change-induced stresses.

Most of the responses mentioned above are medium and long term, and consequences of changes in gene expression. Previous transcriptome analyses revealed how grapevines respond to water deficit at transcriptomic level [15, 16]. Further studies showed different strategies and reactions to different biotic and abiotic stresses at transcript level [16, 18–21], which pointed to the *BURP* family, *MYBs*, or genes related to the phenylpropanoid pathway, as biomarkers of water deficit stress in *V. vinifera* L. [16, 22–24].

The use of specific rootstocks, as it was previously employed for inducing resistance to Phylloxera, may prevent further damages caused by water deficit periods or increased salinity [18, 21, 25]. However, a few studies go through the possible scion–rootstock interaction under abiotic stresses [18, 20, 26]. This is especially relevant for near-isohydric cultivars like *Callet,* which exhibit better tolerance to water deficit when grafted onto the same rootstock, compared to anisohydric cultivars like *Merlot* under similar conditions. Some studies worked on the possible gene expression modification using RNA-seq technology in roots in front of phosphate or nitrogen availability under different scions, revealing possible influences in rootstock by scion [18]. Furthermore, the cross-talk between rootstock and scion is guided by some factors like hormones or miRNAs [27]. However, and despite its importance in current climate change scenario, the interaction between the cultivar and rootstock during water deficit stress at physiological, metabolic, and transcriptional levels is unknown.

Consequently, a comprehensive vision of the changes in different cultivars classified as near-isohydric and anisohydric grapevines, considering the potential effects given by the rootstock, would provide us major facilities to select and improve vineyard management by the use of genotypes more tolerant to low water availability. This general point of view needs to know the changes at physiological, metabolic, and transcriptomic level, which should contribute to better understand the differences among isohydric and near-isohydric plants. The application of these methodologies allows the study of transcription factors such as *MYB* or *BURP*, which are key for water deficit tolerance and highlight the importance of ABA in the response to water deprivation in grapevines. Furthermore, the exploration through the relationship between scion and rootstock under water deficit stress is critical. The principal aim of this study is to understand the differences between near-isohydric cultivars like *Callet*, and near-anisohydric cultivars like *Merlot*, grafted in rootstocks, at physiological, metabolic, hormonal, and transcriptomic levels under water deficit stress. Indeed, this study also aims to explore scion–rootstock relationship under water scarcity.

## Results

### Physiological measurements

**Figure 1:**
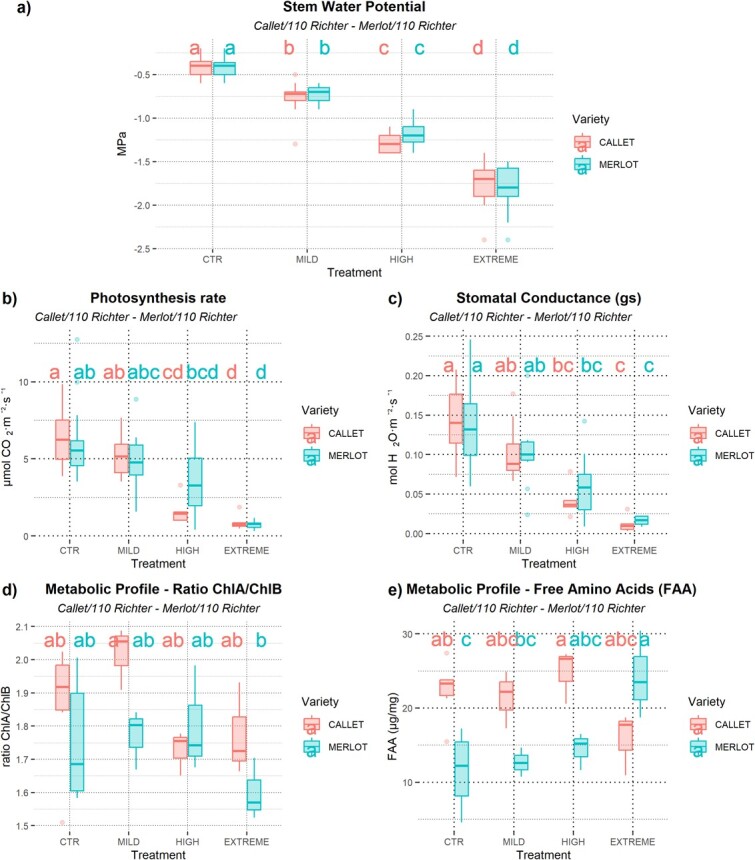
Boxplot of physiological state measures of the plants describing: a) Stem Water Potential expressed in MPa, b) net photosynthetic activity (expressed as μmol CO_2_·m^−2^·s^−1^), c) stomatal conductance (expressed as mol H_2_O·m^−2^·s^−1^), d) Metabolic profile of Chl A/Chl B ratio expressed in units, and e) Metabolic Profile of Free Amino Acid measures expressed in μg/mg dry leaf tissue; in Mild, High, and Extreme conditions on 2-year study (2020 and 2022) for *Callet/110 Richter* and *Merlot/110 Richter*. Different letters indicate significant differences between combinations of variety and treatment (*P* < .05) according to Tukey's *post hoc* test.

Stem water potential showed no significant differences between *Callet/110 Richter* and *Merlot/110 Richter* cultivars within the same treatment. However, significant differences were observed between the different water deficit treatments, indicating similar water status across both cultivars (Fig. 1a). Physiological measurements revealed significant differences between control and water deficit stages in both cultivars, with a clear trend of reduced photosynthetic activity and stomatal conductance rates (Fig. 1b and c). Notably, photosynthesis exhibited distinct behavior: *Merlot/110 Richter* showed a gradual reduction, while *Callet/110 Richter* experienced a substantial decline as water deficit stress intensified (Fig. 1b and c). Stomatal regulation did not differ significantly between the two cultivars (Fig. 1c). Metabolic profiles showed varying trends in chlorophyll ratio and Free Amino Acid Content between cultivars at each water deficit level, although these results lacked statistical significance across treatments (Fig. 1d ande).

### Primary metabolite analysis

**Figure 2:**
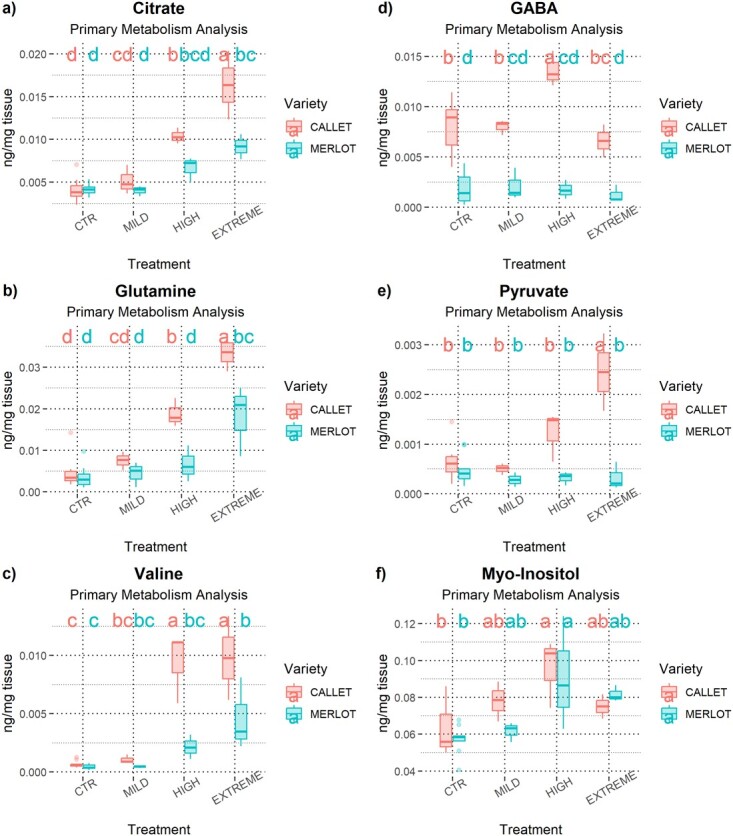
Boxplot of metabolomic results for key primary metabolites: a) Citrate Concentration (in ng Citrate/mg dry tissue), b) GABA Concentration (in ng GABA/mg dry tissue), c) Glutamine Concentration (in ng Glutamine/mg dry tissue), d) Pyruvate Concentration (in ng Pyruvate/mg dry tissue), e) Valine Concentration (in ng Valine/mg dry tissue), and f) and Myo-Inositol Concentration (in ng Myo-Inositol/mg dry tissue) on Mild, High, and Extreme conditions in the year 2020 for *Callet/110 Richter* and *Merlot/110 Richter*. Different letters indicate significant differences between combinations of variety and treatment (*P* < .05) according to Tukey's *post hoc* test.

The primary metabolite analysis revealed further differences between *Callet/110 Richter* and *Merlot/110 Richter*. Of the total of 24 principal metabolites considered, some displayed varying concentrations across water deficit conditions (Supplementary Tables S1 and Table S3). Significant differences and trends were observed in metabolites directly related to water deficit stress response, such as Citrate, GABA, Glutamine, Pyruvate, Valine, or Myo-inositol (Fig. 2 and Supplementary Tables S1 and Table S3).

### Hormonal analysis

**Figure 3:**
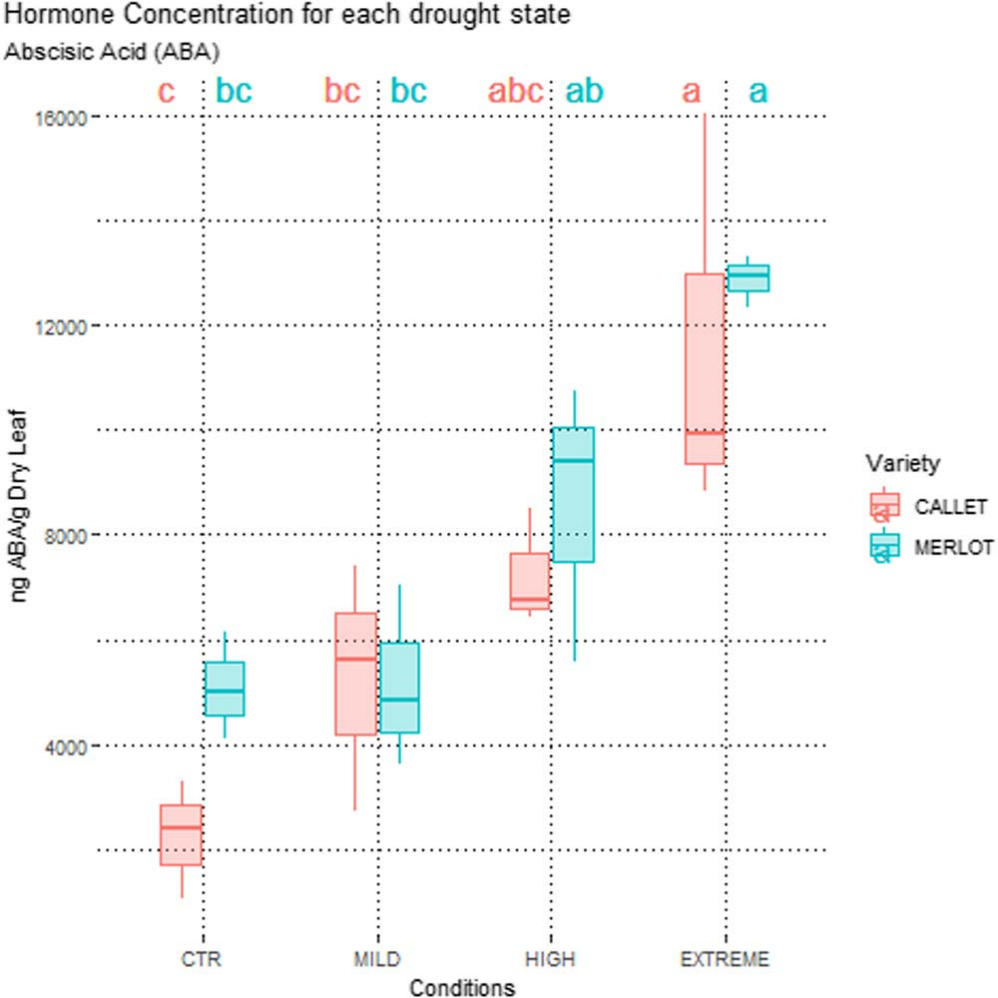
Boxplot of the results obtained at hormonal level for ABA concentration in leaves (expressed in ng ABA/g dry tissue) in 2022 on Mild, High, and Extreme water deficit conditions for *Callet/110 Richter* and *Merlot/110 Richter*. Different letters indicate significant differences between combinations of variety and treatment (*P* < .05) according to Tukey's *post hoc* test.

A low number of significant differences was found in ABA concentrations between the cultivars, but ABA levels increased progressively with water deficit increase (Mild, High, and Extreme) (Fig. 3). Other hormones exhibited varying patterns, with concentrations of Indolacetic Acid (IAA), Jasmonic Acid (JA), and Salicylic Acid (SA) decreasing as water deficit intensified (Supplementary Material 1Fig. S1**)**, defining an increasing pattern between the two cultivars in response to water deficit stress. However, in general, no significant differences in hormone concentrations were observed between *Callet/110 Richter* and *Merlot/110 Richter*.

### Differential expression gene analysis of RNA-seq data

RNA-seq analysis from the years 2020 and 2022 revealed distinct adaptive strategies in *Callet/110 Richter* and *Merlot/110 Richter*, with varying gene expression in roots and leaves depending on water deficit intensity. Due to the limited number of differentially expressed genes (DEGs), the Mild condition was excluded from analysis (all the resultant DEG analyses are available in Supplementary Table S2Tables S1–S8 for all the mentioned comparisons).

**Figure 4:**
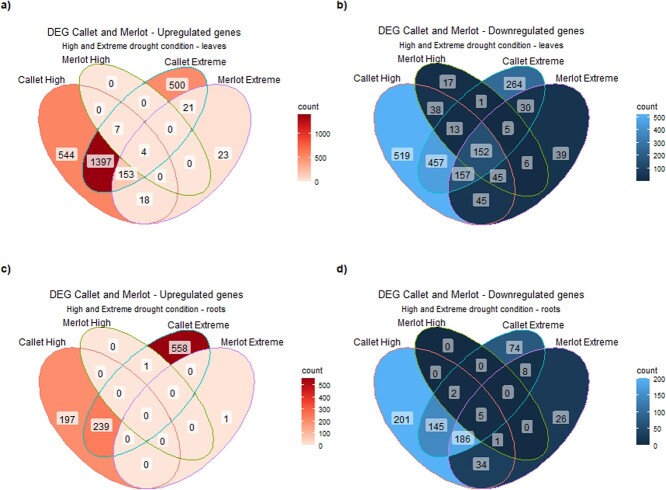
Venn diagram for coincident genes coming from DEG analysis results performed on the 2 years’ data (2020 and 2022), separated at upregulated and downregulated level, for a) *Callet/110 Richter* and *Merlot/110 Richter* upregulated genes in leaves, b) *Callet/110 Richter* and *Merlot/110 Richter* downregulated genes in leaves, c) *Callet/110 Richter* and *Merlot/110 Richter* upregulated genes in roots, and d) *Callet/110 Richter* and *Merlot/110 Richter* downregulated genes in roots.

The Venn diagrams (Fig. 4), compiled data from both years, and examined upregulated and downregulated genes in leaves and roots, show unique water deficit responses in the two cultivars (all Gene Ontology (GO) terms for each coincidence in the Venn diagram are available in Supplementary Table S2Table S9 and their representativity in the total GO universe in Supplementary Material 2Figs S1–S3). This representation provided new insights into how these different genotypes respond to water deficit stress at different levels, highlighting a more prominent stress response in *Callet/110 Richter* compared to *Merlot* and indicating distinct patterns between the two cultivars. In broad terms, *Callet/110 Richter* upregulated a higher proportion of genes in leaves and roots (leaves: 60% upregulated, 39% downregulated; roots: 60% upregulated, 39% downregulated among the identified DEGs) than *Merlot*. Water deficit stress also produced an increase in the percentage of upregulated genes for *Merlot* (leaves: 12% upregulated, 5% downregulated; roots: <0.1% downregulated, 5% upregulated among the identified DEGs in leaves and roots) under water deficit stress (detailed results in Supplementary Table S2Table S10).

The core set of DEGs for each treatment in *Callet/110 Richter* and *Merlot/110 Richter* exhibited common functions related to stress, governing genes associated with ROS, and defense responses in leaves. However, a small overlap in DEGs was identified (156 shared genes in leaves and only 5 genes in roots), pointing to differences in gene regulation, especially in the rootstock component, which become more pronounced as water deficit stress increased.

These differences persisted as water deficit severity increased. In leaves, under High water deficit conditions, *Callet/110 Richter* exhibited significant upregulation of a substantial number of genes related to hormone response and photosynthesis-related genes (2292 genes) while simultaneously downregulating genes associated with light stimulus and growth. In contrast, *Merlot/110 Richter* downregulated 277 genes involved in regulation of cellular processes and growth (Fig. 4a and b, Supplementary Table S2Table S9 and Supplementary Material 2Figs S1–S3). In the roots, *Callet/110 Richter* upregulated 436 genes related to growth, metabolic processes, and ion homeostasis, while downregulating those linked to gene regulation and biosynthesis of components. Nonetheless, *Merlot/110 Richter* displayed minimal root response, with only nine genes (Fig. 4c and d, Supplementary Table S2Table S9 and Supplementary Material 2Figs S1–S3).

When water deficit reached the Extreme state, the gene expression patterns largely mirrored those seen in the High water deficit stage. In *Callet/110 Richter* leaves, 2083 genes were upregulated, focusing on photosynthesis and response to abiotic stimulus, while 1079 genes related to cell communication, catabolic processes, and regulation of gene silencing by miRNAs and protein ubiquitination were downregulated (Fig. 4a and b). Meanwhile, *Merlot*/*110 Richter* leaves showed 277 genes upregulated, mainly linked to photosynthesis and radiation response, while 548 genes related to catabolism were downregulated.

In the roots of *Callet/110 Richter*, the response involved upregulating genes associated with ion transport and growth while downregulating genes linked to other metabolic processes like the regulation of gene expression and macromolecule synthesis (Fig. 4c and d). In contrast, *110 Richter* rootstocks grafted with Merlot did not exhibit any specific regulated functions (Fig. 4c and d) (all the GOs related to these stages are available in Supplementary Table S2Table S9 and Supplementary Material 2Figs S1–S3).

Indeed, the Principal Component Analysis (PCA) performed on the same comparisons was complementary to the Differential Gene Expression (DEG). The PCA identified genes related to response to ABA (e.g. Vitvi01g00556, Vitvi06g01696, Vitvi08g02226), response to water deprivation, and response to light stimulus (see complete gene list in Supplementary Table 3 Table 11). This analysis revealed a separation between the different water deficit levels in the leaves of *Callet/110 Richter* and *Merlot/110 Richter* (Supplementary Material 2Fig. S4) despite the year effect observed in the analysis for 2020 and 2022 (see year effect in Supplementary Material 2Fig. S4 and S5, and Supplementary Material 3).

In roots (Supplementary Material 2Fig. S5), the irrigated plants showed similar distribution in the PCA, with no differences between *Callet* and *Merlot* cultivars at gene expression level. Nonetheless, the distribution changed under increased water deficit stress, resulting in a clear separation between *Callet-* and *Merlot-*grafted plants (Supplementary Material 2Figs S4 and S5, Supplementary Table S3Tables S1–S12 for the whole PCA loadings and function and identification of the most representative loadings in Supplementary Table S3Table S13). The co-expression gene analysis on the whole samples revealed different groups of genes with similar expression pattern depending on the water deficit condition, containing genes related to the stress detoxification and response to ABA in leaves and roots (Supplementary Material 2**,**Figs. S6 to S9, genes by cluster for each condition in Supplementary Table S3Table S14 and principal functions for each cluster in Supplementary Table S3Table S15).

**Figure 5:**
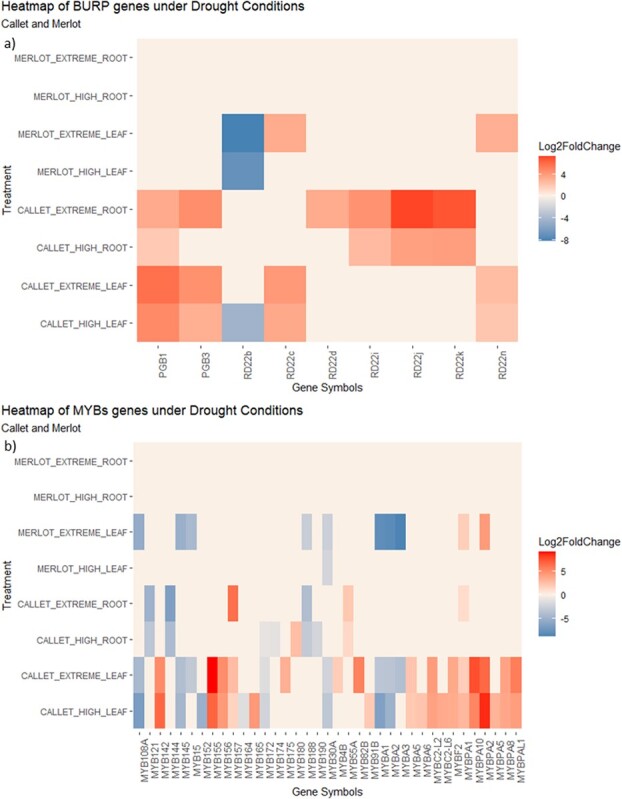
Heat maps of the most represented families in ABA response genes, like a) *BURP* genes and b) *MYBs* genes, represented by Log2foldChange, on leaves and roots of *Callet/110 Richter* and *Merlot/110 Richter* plants submitted to different water deficit stages (High and Extreme).

The DEG analysis results also revealed key genes related to the response to water deprivation, including those involved in crucial pathways like stilbene synthesis or cell wall metabolism (see the most important gene families in Supplementary Material 1Figs S2–S5). Additionally, the analysis revealed DEGs related to one of the most important families related to water deficit stress described in the literature—the *BURP* gene family (Fig. 5a and b). In the gene selection search performed on the DEG results for *Callet/110 Richter* and *Merlot/110 Richter*, gene families such as *BURP* (Fig. 5a) and *MYB* (Fig. 5b) exhibited differences in gene expression levels among other principal families related to water deficit stress (in Supplementary Material 1Figs S2–S5).

Figure 5a illustrated the differences in gene expression for the *BURP* DEGs identified at each water deficit stage, revealing a clear difference in genes like *VvRD22b* (Vitvi11g00340), *VvRD22d* (Vitvi04g00345), or *VvRD22k* (Vitvi04g01870) between *Callet/110 Richter* and *Merlot/110 Richter* in leaves and roots. The modulation of gene expression was higher in *Callet/110 Richter* than in *Merlot/110 Richter*. Furthermore, the gene expression of *MYB* (Fig. 5b) genes like *VvMYB15* (Vitvi05g01733), *VvMYBA1* (Vitvi02g01019), or *VvMYBA2* (Vitvi02g01015) also revealed differences between *Callet/110 Richter* and *Merlot/110 Richter* leaves.

## Discussion

Water deficit treatment significantly impacted stomatal conductance and photosynthesis, with effects becoming more pronounced under higher water deficit stress (Fig. 1b and c), in both cultivars. However, results indicate distinct responses to water deficit between *Callet/110 Richter* and *Merlot/110 Richter*, particularly in these key physiological aspects. Notably, the reduction in carbon fixation rates was more severe in *Callet/110 Richter* under high water deficit conditions compared to *Merlot/110 Richter*. However, under extreme water deficit conditions, *Callet/110 Richter* maintained higher photosynthetic rates than *Merlot/110 Richter*, demonstrating better water use efficiency (WUEi) (Supplementary Material 1, Fig. S6). This preservation of carbon assimilation under water deficit stress, despite stomatal closure regulation, is crucial for survival [5, 14, 28].

Although physiological alterations facing water deficit were notable, metabolite profile analysis also revealed relevant changes between cultivars at different water stress levels (Fig. 2, Supplementary Material 1Fig. S7). Variations among *Callet/Merlot* in photosynthesis-related metabolites supported previous observations (Fig. 1; Supplementary Material 1Fig. S7) explaining the possible differences in photosynthetic rates due to increased tolerance to high illumination in plants [29], while the differential free amino acid content suggested additional pathways affected by water deficit, possibly indicating protein degradation or alterations in phenolic compounds. The alteration of phenolics, involved in reducing oxidative damage, could reflect a differential regulation of metabolic pathways to minimize the impact of water deficit stress [30], becoming higher in *Callet/110 Richter* than *Merlot/110 Richter* (Supplementary Material 1Fig. S7).

Further analysis of primary metabolites revealed distinct responses to water deficit stress and differences between genotypes, possibly attributed to variations in ABA sensitivity. For instance, the different patterns observed in metabolite concentrations such as citrate, GABA, glutamine, pyruvate, and valine under increasing water deficit stress (Fig. 2) suggested different metabolic responses between *Merlot/110 Richter* and *Callet/110 Richter* [17, 31–33].

As expected, the increasing water deficit stress resulted in an increased ABA concentration, in a similar way in leaves of both cultivars (Fig. 3). Surprisingly, both cultivars had similar ABA concentrations at the same stem water potential (Fig. 1a), suggesting that additional factors, beyond ABA, play a crucial role in grapevine response to water deficit and acclimation [16, 34]. This multimodal response was reflected in different concentrations of other metabolites analyzed as in the case of the myo-inositol, higher in *Callet/110 Richter* than in *Merlot/110 Richter*, and acts as an osmolyte, reducing water loss during water deficit stress, or the essential role of GABA (see myo-Inositol or GABA differences, among other primary metabolites in Fig. 2) and its metabolism regulation for ROS reduction or Ca^2+^ signaling under water deficit stress [17, 34].

Transcriptomic analysis further elucidated the mechanisms to water deficit stress response. PCA and differential gene expression (DEG) analysis identified key genes associated with water deficit tolerance, including aquaporins, ABA-responsive genes, and genes involved in flavonoid biosynthesis [35–37]. *Callet/110 Richter* exhibited a more specific transcriptomic response compared to *Merlot/110 Richter*, particularly in terms of plant development and detoxification pathways (Supplementary Material 2Figs S1–S3). *Callet/110 Richter* and *Merlot/110 Richter* also showed different strategies when comparing root and leaves transcriptomes, considering the differences at species level (*Callet and Merlot* as *V. vinifera* and *110 Richter* as an interspecies hybrid *Vitis berlandieri* × *Vitis rupestris*) (Fig. 4). The differential response was also evident in candidate gene analysis, where genes related to stress response, secondary metabolite production, and root growth showed distinct expression patterns between the cultivars (Fig. 5 and Supplementary Material 1Figs S2–S5). Notably, genes related to ABA signaling, such as members of the *BURP* and *MYB* families, exhibited differential expression, potentially contributing to the observed differences in water deficit tolerance [15, 22–24].

In leaves, *Callet/110 Richter* exhibited an upregulation of genes associated with photosynthesis under high water deficit conditions, whereas *Merlot/110 Richter* displayed a less resilient response, sharing some of the expressed genes with *Callet/110 Richter*. This upregulation of photosynthesis-related genes during water deficit stress aligns with studies indicating a protective mechanism for PS/thylakoid damage caused by water deficit stress [38], which could be linked to the observed reduction in photosynthesis and differences in chlorophyll levels (Fig. 1d, and Supplementary Table S2Table S9). Notably, *Merlot/110 Richter* showed similar gene expression patterns in photosynthesis-related genes and radiation response only under extreme water deficit conditions, contrasting with *Callet/110 Richter*, which exhibited modulation at high water deficit conditions (Fig. 1b, d and Supplementary Table S2Table S9). Conversely, in roots, *Callet/110 Richter* plants responded by upregulating genes involved in growth, metabolic processes, and ion homeostasis, while *Merlot/110 Richter* plants displayed a comparatively lower transcriptomic response.

The differences in gene expression were further supported by the identification of candidate genes associated with water deficit stress response. Gene expression patterns of families such as *BURP*, *MYB*, and those related to the Flavonoid Pathway, sugar transportation, and lignin [22, 23] differed between the two cultivars, with *Callet/110 Richter* showing coordinated responses in both leaves and roots (Supplementary Material 1Figs S2–S5). The *BURP* family, e.g. plays a crucial role in understanding the response of water deficit-tolerant grapevines to varying degrees of water deficit stress. In *Callet/110 Richter* the variation in gene expression of some genes in leaves was followed by a regulation of gene expression in roots similarly, e.g. in genes related to sugar transportation like *VvHT4* (Vitvi16g00479) or *VvHT5* (Vitvi05g00468), related to sugar uptake in extracellular environment in order to reduce the activity into the plant [39, 40], or in genes related to the flavonoid pathway, like *VvCHS2* (Vitvi14g01449), *VvCHS3* (Vitvi05g01044), and *VvLDOX1* (Vitvi02g00435), previously described as possible related genes in response to water deficit in grapes and leaves [22, 24, 41]. The importance of the *BURP* family in water deficit could be essential to understand how water deficit-tolerant grapevines could react to water deficit stress under different stages of water privation, and the results showed similar gene expression patterns in the DEGs detected, directly related to ABA, like the *VvRD22b* (Vitvi11g00340) downregulation and the *VvRD22c* (Vitvi04g00342) upregulation (Fig. 5). *RD22* (Response to Dehydration 22) superfamily is involved in water deficit stress response, encoding structural proteins located in the apoplast and cell wall [23, 42].

The involvement of the *BURP* family genes in the response to abiotic stress in grafted plants presents a new window for exploring their role in scion–rootstock communication. ABA's influence on the scion could potentially enhance responses in the roots, such as root lignification and regulation of root elongation (Fig. 5, Supplementary Table S2Table S9). For instance, the regulation of genes related to lignin, such as *VvLAC20* (Vitvi13g00117), are crucial for mitigating water loss by increasing impermeability, but reducing root growth (Fig. 5b and Supplementary Material 1Fig. S3) [43].

Within the *MYB* family, a general overexpression tendency has been observed comparing *Callet/110 Richter* with *Merlot/110 Richter* in leaves and roots (Fig. 5). This finding contrasts with previous studies such as Savoi *et al.*, 2017 [44] or Galbiati *et al.*, 2011 [45], highlighting the complexity of grapevine response to water deficit and its variation across different genotypes. Indeed, the DEG analysis showed different regulation in genes like *VvMYB15* (Vitvi05g01733), *VvMYBA1* (Vitvi02g01019), *VvMYBA2* (Vitvi02g01015), or *VvMYBA3* (Vitvi02g01024), which are implicated in stress response and secondary metabolite production [15, 16, 22, 24]. For instance, *VvMYB15* is associated with the expression of genes involved in stilbene or flavonoid production, which alleviate abiotic stresses by regulating genes from the Flavonoid Pathway like *VvLDOX1* (Vitvi02g00435) or *VvUFGT1* (Vitvi16g00156), as observed in the differential expression between *Callet/110 Richter* and *Merlot/110 Richter* (Fig. 5 and Supplementary Material 1Fig. S2) [22, 24], supporting the previous metabolic observations and probably related to the mitigation of oxidative damage.

Moreover, the phenylpropanoid pathway is implicated in the response, with a general upregulation of families involved in water deficit stress response, such as *C4H* (Supplementary Material 1Fig. S4) [46]. Despite the substantial evidence implicating these gene families, the intricate network among them and their regulation of other unknown genes is likely mediated by transcription factor families like *WRKYs* and *bZIPs*, also related to ABA. These transcription families are regulated in response to water deficit stress being higher the regulation in *Callet/110 Richter* than in *Merlot/110 Richter* in transcription factors like *VvbZIP52* (Vitvi19g00147), reported in rice as modulator of water deficit stress response (Supplementary Material 1Fig. S5) [47–49].

The coordination between scion and rootstock also appeared crucial in water deficit adaptation. When *Merlot/110 Richter* was used as the scion and *110 Richter* as the rootstock, a possible lack of coordination was observed, with lower gene expression levels in leaves and roots compared to *Callet/110 Richter* (Figs 4, 5, Supplementary Material 1Figs S2–S5 and Supplementary Material 2Fig. S5). This lack of coordination could be attributed to the cross-talk between the cultivar and rootstock, evidenced by differential expression of genes related to miRNA regulation, which was absent in *Merlot/110 Richter*, probably caused by the cross-talk between the cultivar *vinifera* and the American grapevine rootstock [18, 20, 27, 50]. This effect could be explained by the origin of the reduction of water availability, which is produced into the roots and the extension to the aerial part that induces ABA production (Fig. 3) [18, 50].

This interaction highlights the importance of effective coordination between the scion and rootstock in water deficit adaptation, revealing an intricate maze of responses affecting the expression of key genes related to the response to ABA in grapevine. This cross-talk could be initiated by the increase in ABA production in leaves in response to reduced water availability in roots, subsequently communicating the water deficit signal to the rest of the binomial plant/scion [26]. The sensitivity to ABA in *Callet/110 Richter* facilitated this adaptation process, leading to physiological adjustments such as reduced photosynthesis rates (Fig. 1). Additionally, it triggered further adaptations related to primary and secondary metabolite production and root elongation, as evidenced by transcriptomic responses. However, the low sensitivity to ABA in *Merlot/110 Richter* would limit this response, resulting in a reduced specificity of gene expression and metabolite analysis, particularly in roots under high drought conditions [16, 51].

The findings underscore the multifaceted nature of grapevine water deficit tolerance, highlighting the interaction between genotype and the communication between scion and rootstock. Specifically, our findings suggest that ABA sensitivity and other described actors such as primary metabolites played a crucial role in facilitating a more comprehensive response in *Callet* compared to *Merlot*, both grafted with *110 Richter* rootstocks. Notably, the differential responses observed in gene expression and metabolite profiles between *Callet/110 Richter* and *Merlot/110 Richter* underscore the intricate regulatory networks involved in water deficit response and the consequent resilience to arid environments. The impaired communication between scion and rootstock caused by a possible deficient ABA sensitivity in *Merlot/110 Richter* reduced the capability of the plant to adequately respond to stress, forcing a non-specific response to water deficit instead of the specialized response exhibited by *Callet/110 Richter*.

Furthermore, the identification of candidate genes associated with water deficit stress response, such as those in the *BURP* and *MYB* families, sheds light on potential molecular markers for water deficit tolerance [22, 23]. Understanding rootstock–scion interactions is essential for developing strategies to mitigate the impact of water deficit on viticulture and ensuring the resilience of grapevine crops in the face of changing environmental conditions, being absolutely necessary to explore new optimal combinations of scions and rootstocks for a better stress tolerance [18, 20, 26, 52].

## Materials and methods

### Plant material, growth conditions, and treatments

**Figure 6:**
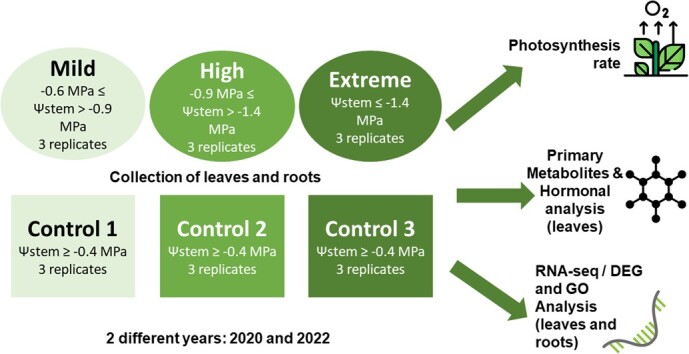
Experimental design on greenhouse conditions at three different water deficit levels depending on the hydric potential of leaves (Mild: −0.6 MPa ≤ Ψ_stem_ ≤ −0.9 MPa; High: −0.9 MPa ≤ Ψ_stem_ ≤ −1.4 MPa; Extreme: Ψ_stem_ ≥ −1.4 MPa) on 2-year study (2020 and 2022).

The experiment was performed at the Center for Plant Biotechnology and Genomics (CBGP UPM-INIA/CSIC) in Madrid, Spain, over two different years (2020 and 2022). The study involved two different grapevine cultivars: *Callet*, a local cultivar from Balearic Island, characterized as near-isohydric, and *Merlot*, a commercial anisohydric cultivar coming from France. A total of 144 plants (72 plants/year) aged 2 years old grafted on *110 Richter* rootstocks for each cultivar cited below were used in the performed experiment. These plants were bought from a commercial plant provider, Vitis Navarra, from Navarra, Spain. The experiment was carried out in greenhouse conditions (16 h light–8 h darkness, with temperature intervals of 16°C–25°C). Plants were grown in cylindrical pots with 21.5 cm diameter and 20.5 cm height, with drainage provided and a substrate mix of 70% peat–30% sand. Each plant was pruned to maintain a single shoot for uniformity. Water deficit stress was imposed, stopping watering when the plants reached out the phenological stage 16–17 [53]. Three different stress levels were defined considering the stem water potential (Ψ_stem_): Mild (−0.6 MPa ≤ Ψ_stem_ > −0.9 MPa), High (−0.9 MPa ≤ Ψ_stem_ > −1.4 MPa), and Extreme (Ψ_stem_ ≤ −1.4 MPa) [54]. Nine plants at each condition and cultivar were sampled and compared to controls (with irrigation, Ψ_stem_ > −0.6 MPa) when the plants reached the expected water stem potential at each case in three different days (see complete collected samples for each condition in Supplementary Table S1). For the determination of each stage, Ψ_stem_ were determined by Scholander chamber (Model 1009, PMS Instruments, Oregon, USA) as in Bota et al. (2016) [4] using the sixth to seventh last leaf. The four last leaves at the end of the shoot and a root portion for each year/condition/control were sampled, flash-frozen with liquid nitrogen, and stored at −80 °C until use (Fig. 6).

### Physiological measurements of the plants

Gas exchange measurements, net photosynthetic rate, and stomatal conductance were measured in the fifth leaf from the apex of the shoot using an open gas exchange system LI-COR 6400 XL (LI-COR Biosciences Inc., USA) on 2-cm^2^ leaf, under 390-ppm CO_2_ concentration [4], and 1000-μE light intensity for greenhouse conditions. The measures were expressed as μmol CO_2_·m^−2^·s^−1^ for the CO_2_ fixation units and mol H_2_O·m^−2^·s^−^1 for the stomatal conductance. Provided values averaged the measurements of nine plants. The intrinsic Water Use Efficiency index (WUEi) was calculated using the ratio between net photosynthesis and the stomatal conductance of the plants, and it was expressed as μmol CO_2_·mol H_2_O^−1^.

Additionally, in order to describe the physiological state for all the plants corresponding to these water deficit states, a metabolic biomarker profile was performed in the second year (2022) to explore the differences in response to water deficit stress in grapevine following the procedure described by López-Hidalgo *et al.*, 2021 [55] and starting from 10 mg of lyophilized leaves samples.

### Primary metabolite and hormonal level analysis

Regarding to the metabolism study of the response to water deficit in scions *Callet* and *Merlot,* two different analyses were performed. The first analysis was performed on the samples coming from 2020, in order to study the primary metabolite content in *Callet* and *Merlot* leaves. For that, three replicates of leaf samples were lyophilized and a primary metabolite analysis was done following the extraction protocol of Choi *et al.* (2004) [56] coming from 50 mg of dried sample and using 1H-Nuclear Magnetic Resonance (1H-NMR), thanks to the analytical service of CEBAS-CSIC (Centro de Edafología y Biología Aplicada del Segura, Espinardo, Murcia, Spain). A total of 24 principal metabolites were considered in the primary metabolite analysis (see complete list in Supplementary Table S1Table S3).

Additionally, hormonal levels of 0.03 g of leaf samples coming from the year 2022 from four different hormones (SA, IAA, ABA, and JA) were quantified (Supplementary Material 1Fig. S1) for each cultivar and water deficit condition using Ultra Performance Liquid Chromatography/Mass Spectrometry (UPLC/MS) using the service for hormonal analysis located at the IBMCP (Instituto de Biología Molecular y Celular de Plantas – UPV/CSIC, Paterna, Valencia, Spain).

### Transcriptomic profiles

#### RNA extraction and RNA-seq performance

RNA extraction of three samples per tissue from roots and leaves of the 2 years’ experiments was performed according to Reid *et al.* (2006) [57]. Isolated RNA were sequenced at Macrogen Inc., South Korea, using Illumina Hiseq 2000 systems for paired-end sequencing and Strong Specific Libraries, reaching an average of 33 million of sequences per sample with a length of 150 bp.

#### Differential expression genes and gene ontology analysis

A Differential Expression Gene (DEG) analysis was developed in order to process all the obtained sequences in RNA-seq using the total of RNA-seq performed on the 2 years (2020 and 2022). Firstly, a general quality control checking was done on all the samples using FastQC v0.11.8 software and a soft trimming were performed on the samples using Trimmomatic v0.38. After trimming, a second quality checking was done, and a next alignment was performed over all the samples using HISAT2 v2.1.0 and the reference genome of *V. vinifera* L. PN40024 v4.1 INTEGRAPE COST Action [58]. Once the sequences were aligned, a count of sequences was done using the reference genome annotation of the cited version below and featureCounts tool available in Rsubread package v2.4.3, in R software v4.0.3. Finally, the resultant counts were analyzed using two different packages of DEG analysis in order to reach a consortium of DEGs from the results of the two different packages [59]. For that, we used DESeq2 package v1.30.1 and NOISeq package v2.34.0 in the same version of R environment cited below, and only the coincident resultant genes produced by the two packages were to be considered. In order to stablish the comparisons, a principal checking of control counts was done comparing all the controls (Control 1, Control 2, and Control 3) to check possible bias among the collected samples among the 2 years. Because of the non-difference among controls, the analysis was performed using Control 1-Mild, Control 2-High, and Control 3-Extreme. For all the comparisons, the DEG analyses were performed on the whole experimental years (2020 and 2022), in order to reduce possible influence of the environment and to identify the specific water deficit response along the transcriptome. All the source code was published at GitHub (available at: https://github.com/alberto-rodriguezizquierdo/RNAseq_analysis). In order to analyze the functional annotation of the resultant genes, only the genes whose −1 ≥ log2FoldChange ≥ 1 and *P*-value <.05 were considered as DEGs. A GO Analysis was done over all the resultant genes using the package GOStats v2.6. On that genes, a gene search for possible responsible gene candidates was performed in order to identify possible families involved in. For that purpose, a search using the gene IDs obtained before was performed using the Gene Reference Catalog of *V. vinifera* L. [60], with the aim of to study the gene expression of each one and comparing them with the bibliography. Additionally, from the general counts of each sample, a PCA was performed on the overall of the compared conditions, in order to identify the principal variables involved in the differences for each condition. The most important genes according to the loadings representativity on the PCA were annotated manually using the Gramene database and finding analogies in model plants. Furthermore, a K-means analysis was performed on the several conditions in leaves and roots for detecting co-expression networks. The functions for each gene cluster were analyzed using GOStats v2.6.

### Validation of differential expression gene analysis results

Seven genes with differential responses to stress were selected to validate differential expression analysis. These genes reflected key elements discovered in this work, combined with other genes with differential expression previously related to water deficit (BURP, MYBs, phenylpropanoids). RNAs were retrotranscribed employing qScript XLT cDNA SuperMix (Quanta Bio) and oligonucleotides were designed employing primer-blast (Supplementary Table S1Table S2b) and the genome of *V. vinifera* 12X version 1. Quantitative real-time PCR (qPCR) was performed employing SYBR Green 1 Master and the Roche LightCycler 480 thermocycler. The relative expression of the results was calculated by 2^-ΔΔCt^ and normalized using the referent and constitutive gene β-actin for normalization.

### Statistical analysis for physiological, metabolic, and hormonal data

Statistical analysis was performed in physiological (stomatal conductance, photosynthesis, water potential, WUE), metabolomic, and hormonal measures using R environment. In order to perform the analysis, a Kolmogorov–Smirnov normality test was done to check the normality of the data. After that, ANOVA-two way for all the possible comparisons between water deficit stages and between cultivars was calculated on the whole possible comparatives, considering ‘Water deficit treatment’ and ‘Cultivar’ as factors. A *post hoc* analysis was performed using the Tukey HSD (honestly-significant-difference) test on the ANOVA results, considering significant difference when *P*-value <.05.

## Supplementary Material

Web_Material_uhae291

## Data Availability

The data underlying this article are available in the European Nucleotide Archive (ENA) (https://www.ebi.ac.uk/ena/browser/home) and can be accessed with PRJEB55563.
