# Supplementary material for: The scion-driven transcriptomic changes guide the resilience of grafted near-isohydric grapevines under water deficit
Source: Hortic Res. 2024 Oct 23;12(2):uhae291. doi: 10.1093/hr/uhae291 (PMC11789524; doi:10.1093/hr/uhae291)

Supplementary Material 1: The sensitivity to ABA in grapevine scion guides the coordination with rootstock under drought conditions.

Rodriguez-Izquierdo et al., 2024

Spain, 2024

Figure 1: Boxplot of the results of a) Indolacetic Acid (IAA); b) Jasmonic Acid (JA); and c) Salicilic Acid (SA) concentration in leaves (expressed in ng ABA / g dry leaves); on Mild, High and Extreme conditions in 2022 for *Callet/110 Richter* and *Merlot/110 Richter*. Not shared letters indicates significant results between comparisons coming from ANOVA-two way and Tukey post-hoc test.

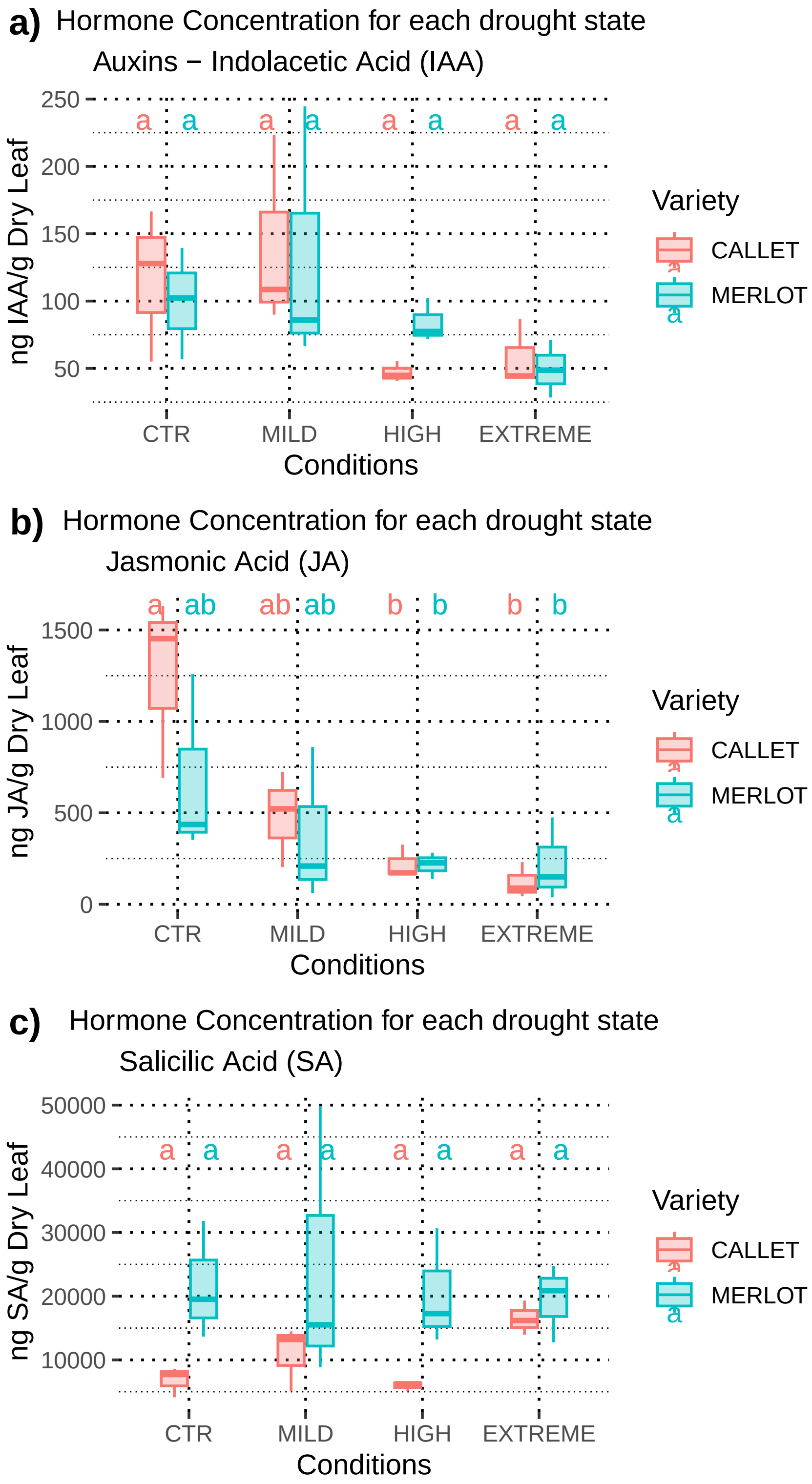

Figure 2: Heatmaps of known DEGs detected in *Callet/110 Richter* and *Merlot/110 Richter* for High and Extreme drought conditions in leaves and roots: a) Flavonoid pathway genes and b) Stilbene pathway genes. The colors represent in blue scale the downregulated genes and in red scale the upregulated genes (expressed at log2foldChange value).

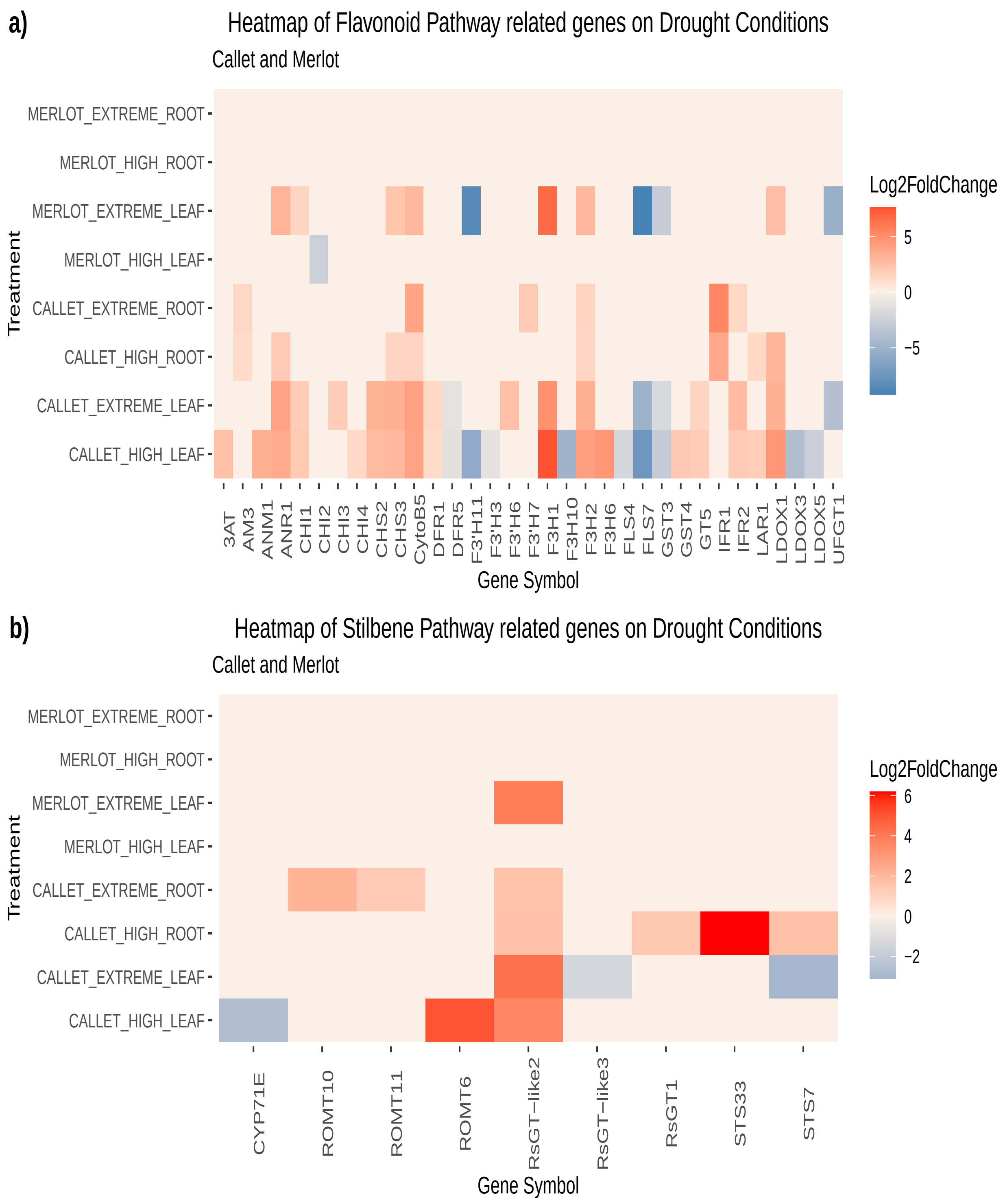

Figure 3: Heatmaps of known DEGs detected in *Callet/110 Richter* and *Merlot/110 Richter* for High and Extreme drought conditions in leaves and roots: a) Lignin pathway genes; and b) Sugar metabolism pathway genes. The colors represent in blue scale the downregulated genes and in red scale the upregulated genes (expressed at log2foldChange value).

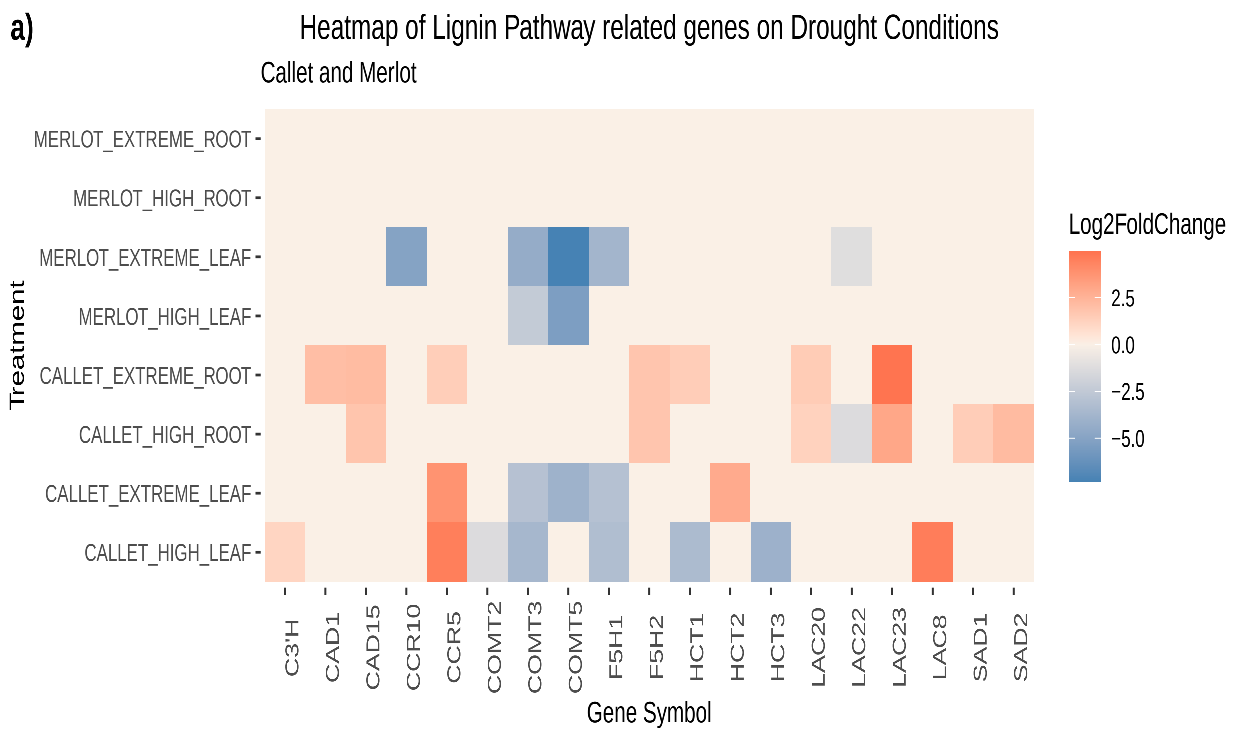

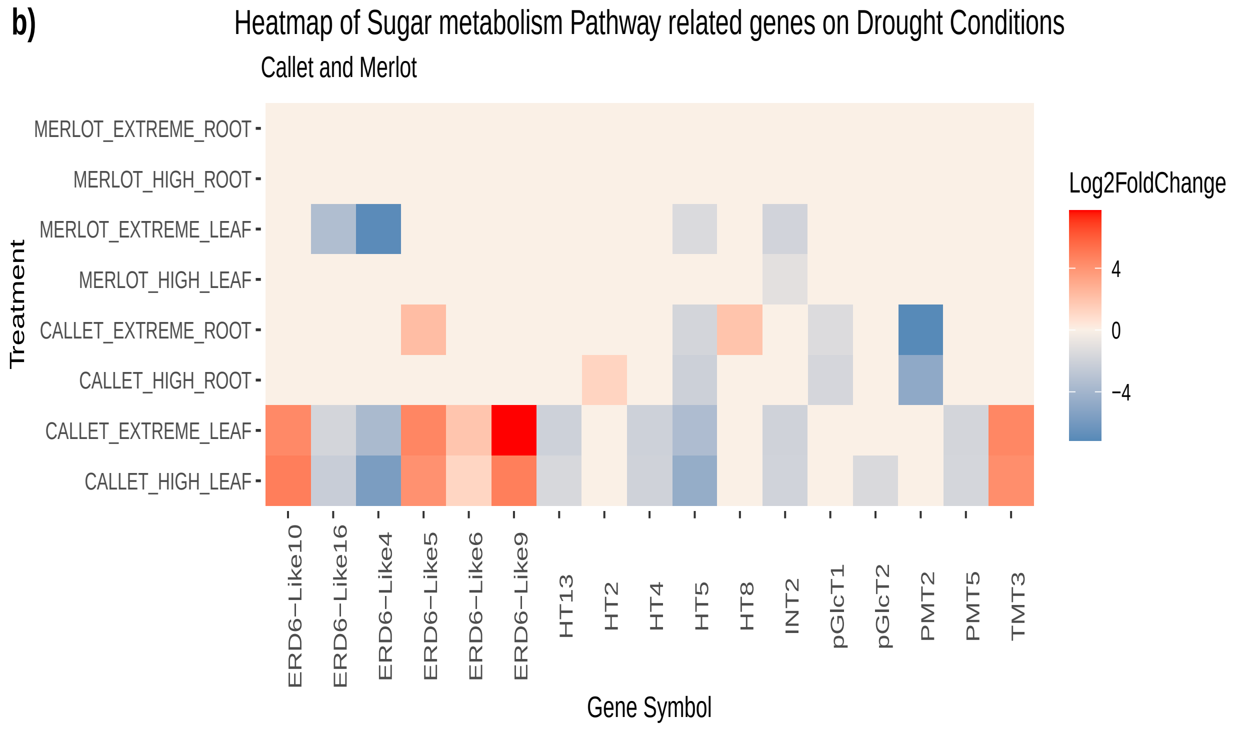

Figure 4: Heatmaps of known DEGs detected in *Callet/110 Richter* and *Merlot/110 Richter* for High and Extreme drought conditions in leaves and roots: a) Phenyl Propanoid pathway genes; and b) WRKYs genes. The colors represent in blue scale the downregulated genes and in red scale the upregulated genes (expressed at log2foldChange value).

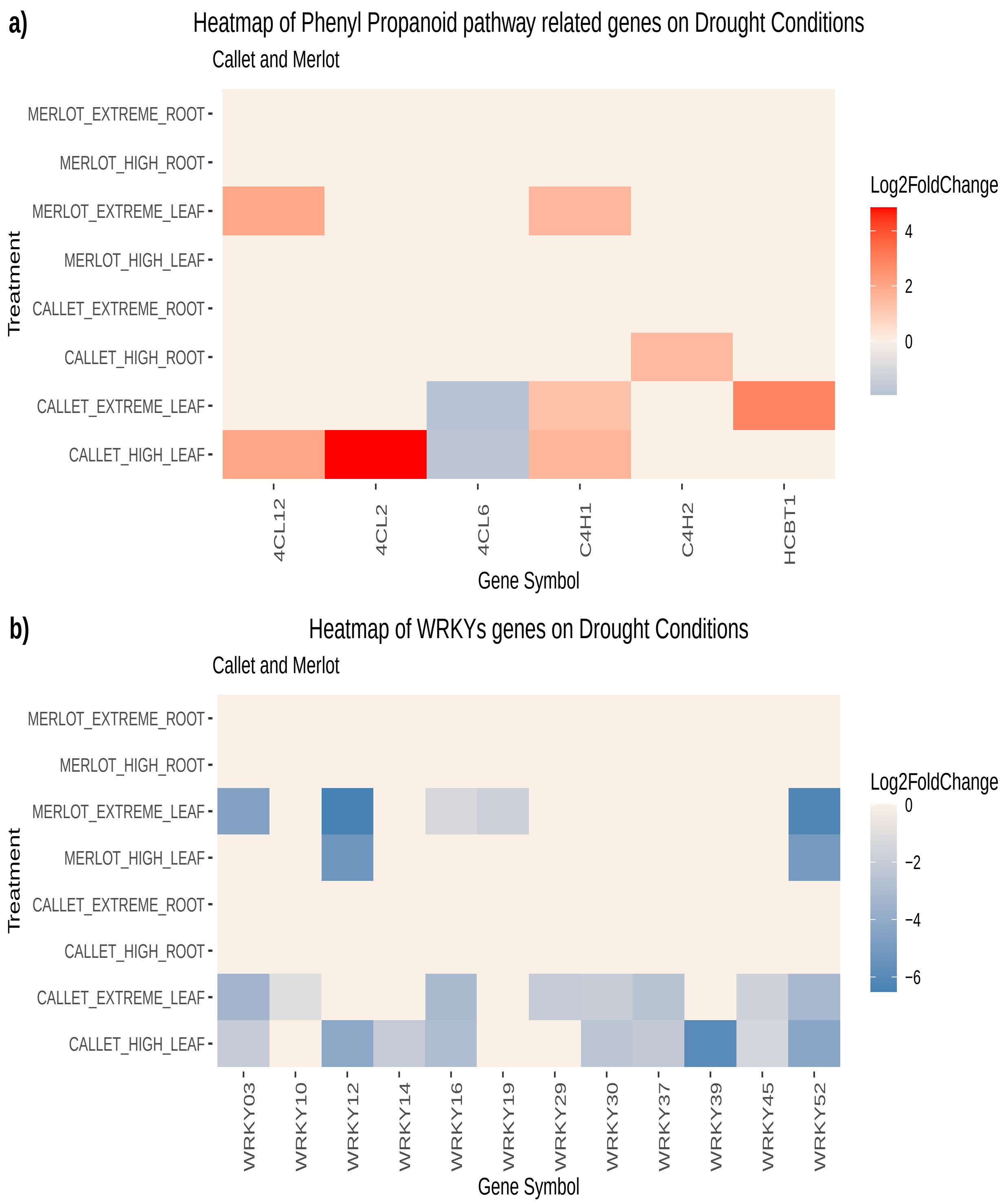

Figure 5: Heatmaps of known DEGs detected in *Callet/110 Richter* and *Merlot/110 Richter* for High and Extreme drought conditions in leaves and roots: a) Caroten pathway genes; and b) bZIPs genes. The colors represent in blue scale the downregulated genes and in red scale the upregulated genes (expressed at log2foldChange value).

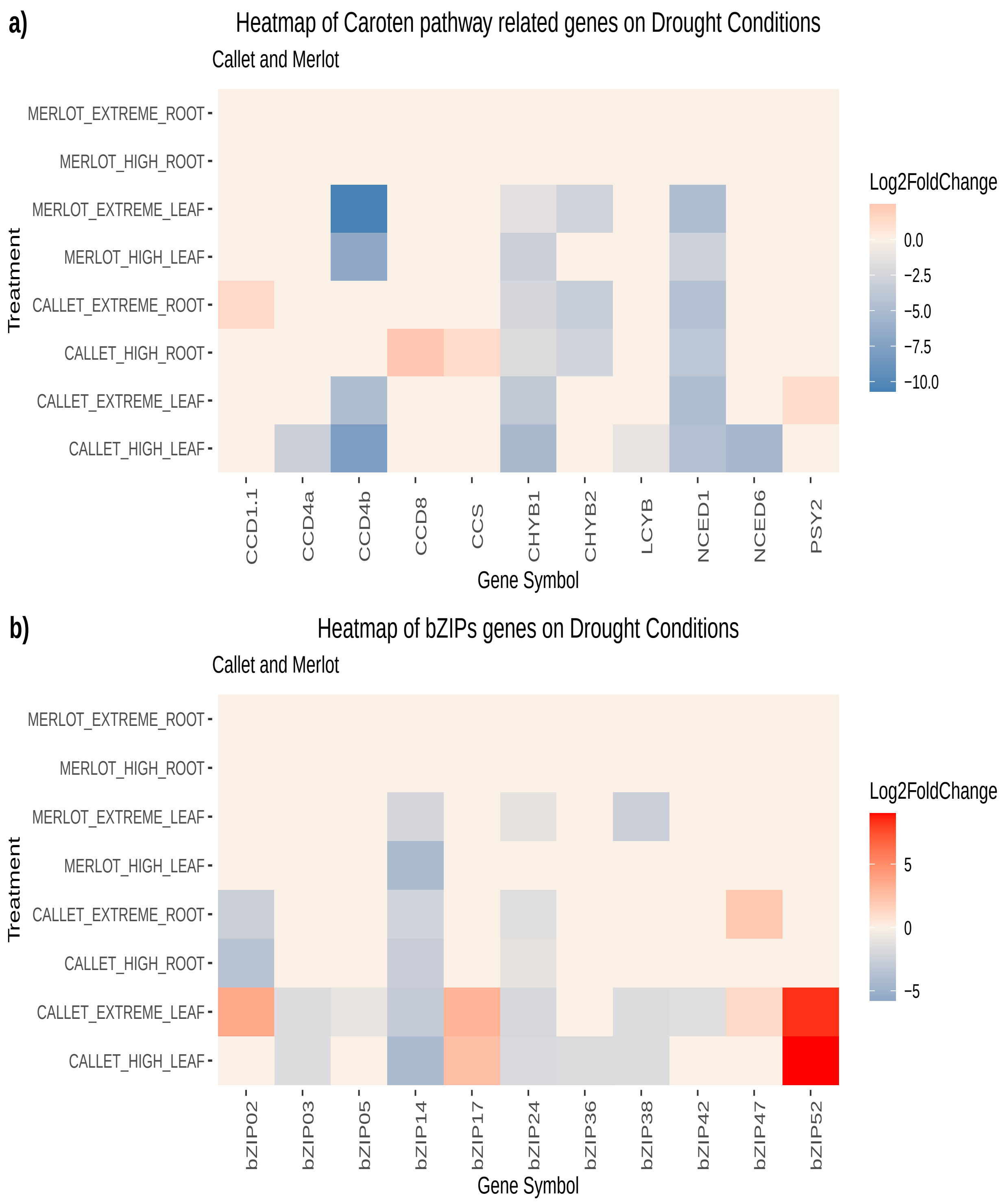

Figure 6: Boxplot of Water Use Efficiency (WUE) expressed in $\mu mol CO_2/mol H_2O$ values for *Callet/110 Richter* and *Merlot/110 Richter* on Mild, High and Extreme conditions in 2020 and 2022. Not shared letters indicates significant results between comparisons coming from ANOVA-two way and Tukey post-hoc test.

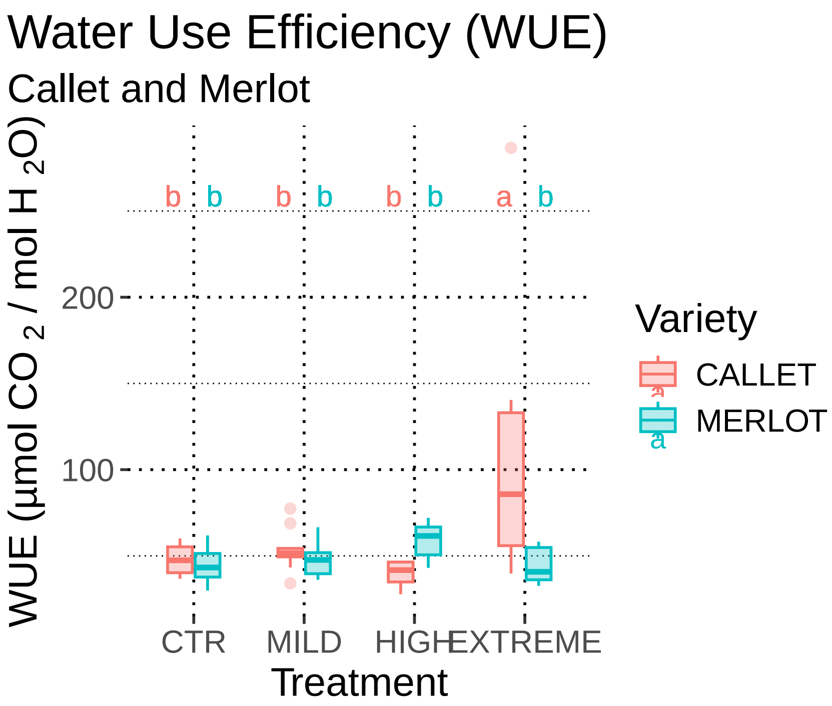

Figure 7: Boxplot of the results for the physiological state of: a) metabolic profile of Chlorophyll A; b) metabolic profile of Chlorophyll B; c) metabolic profile of Total Phenolic Compounds (TPC); d) metabolic profile of Total Flavonols (TFL); e) metabolic profile of Carotenoids (Car); and f) metabolic profile of Starch; all expressed in µg metabolite/mg dry leaves on Mild, High and Extreme conditions in 2020 and 2022 for for *Callet/110 Richter* and *Merlot/110 Richter*. Not shared letters indicates significant results between comparisons coming from ANOVA-two way and Tukey post-hoc test.

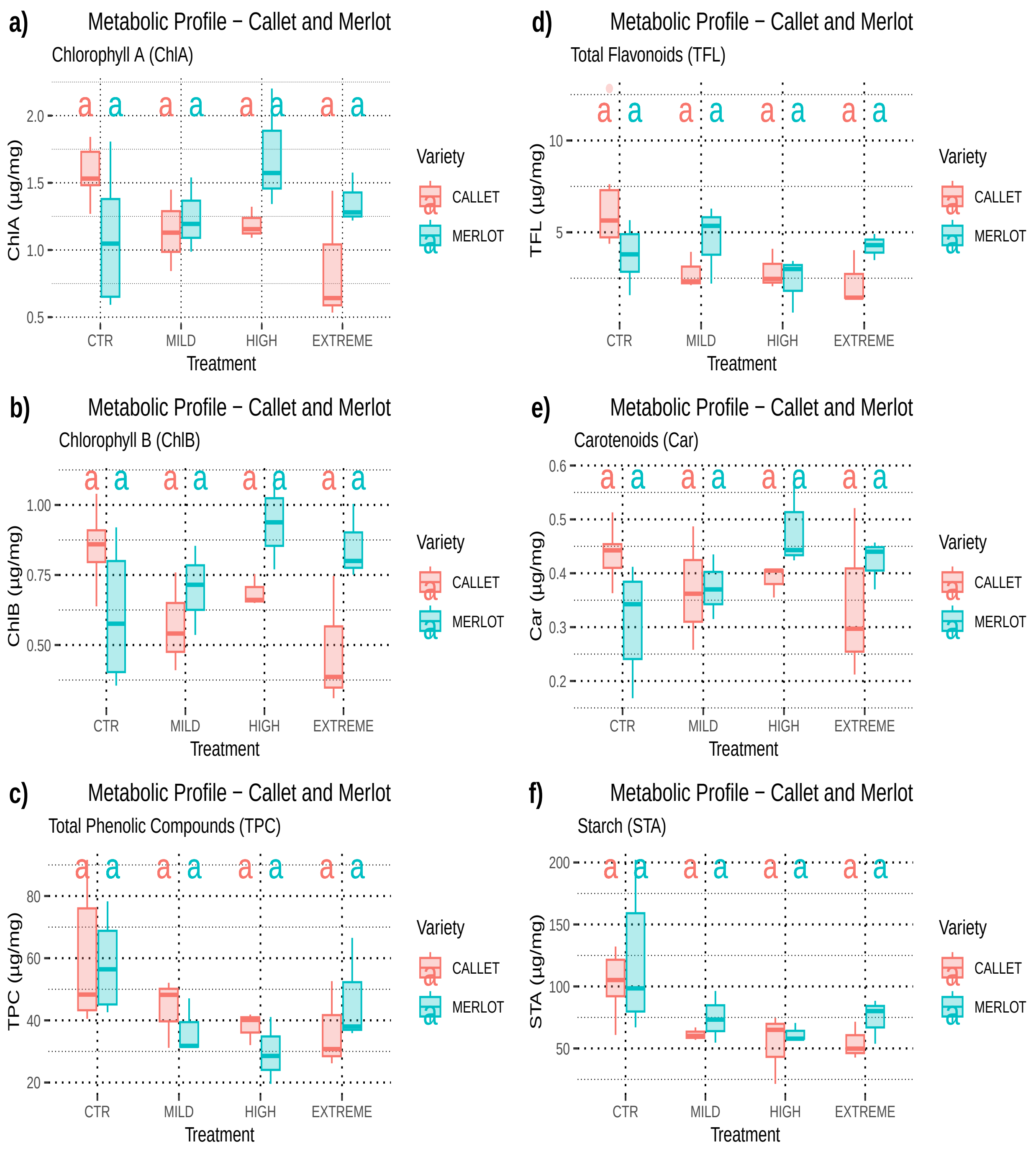

Supplement: Web_Material_uhae291 [file web_material_uhae291.zip › Supplementary_Material_1.pdf]
