# Supplementary material for: The scion-driven transcriptomic changes guide the resilience of grafted near-isohydric grapevines under water deficit
Source: Hortic Res. 2024 Oct 23;12(2):uhae291. doi: 10.1093/hr/uhae291 (PMC11789524; doi:10.1093/hr/uhae291)

Supplementary Material 2: The sensitivity to ABA in grapevine scion guides the coordination with rootstock under drought conditions.

Rodriguez-Izquierdo et al., 2024

Spain, 2024

Figure 1: Principal functions depending on the condition from the Venn Diagram (figure 5 in the article) for: a) upregulated genes in leaves; b) upregulated in roots; c) downregulated genes in leaves; and d) downregulated genes in roots; for *Callet/110 Richter* and *Merlot/110 Richter*. The x-axis represents the number of detected GOs. The bubble color represents each comparison in the Venn Diagram, and the bubble size indicates the percentage of detected GOs for GO Biological Process in the total universe of GOs.

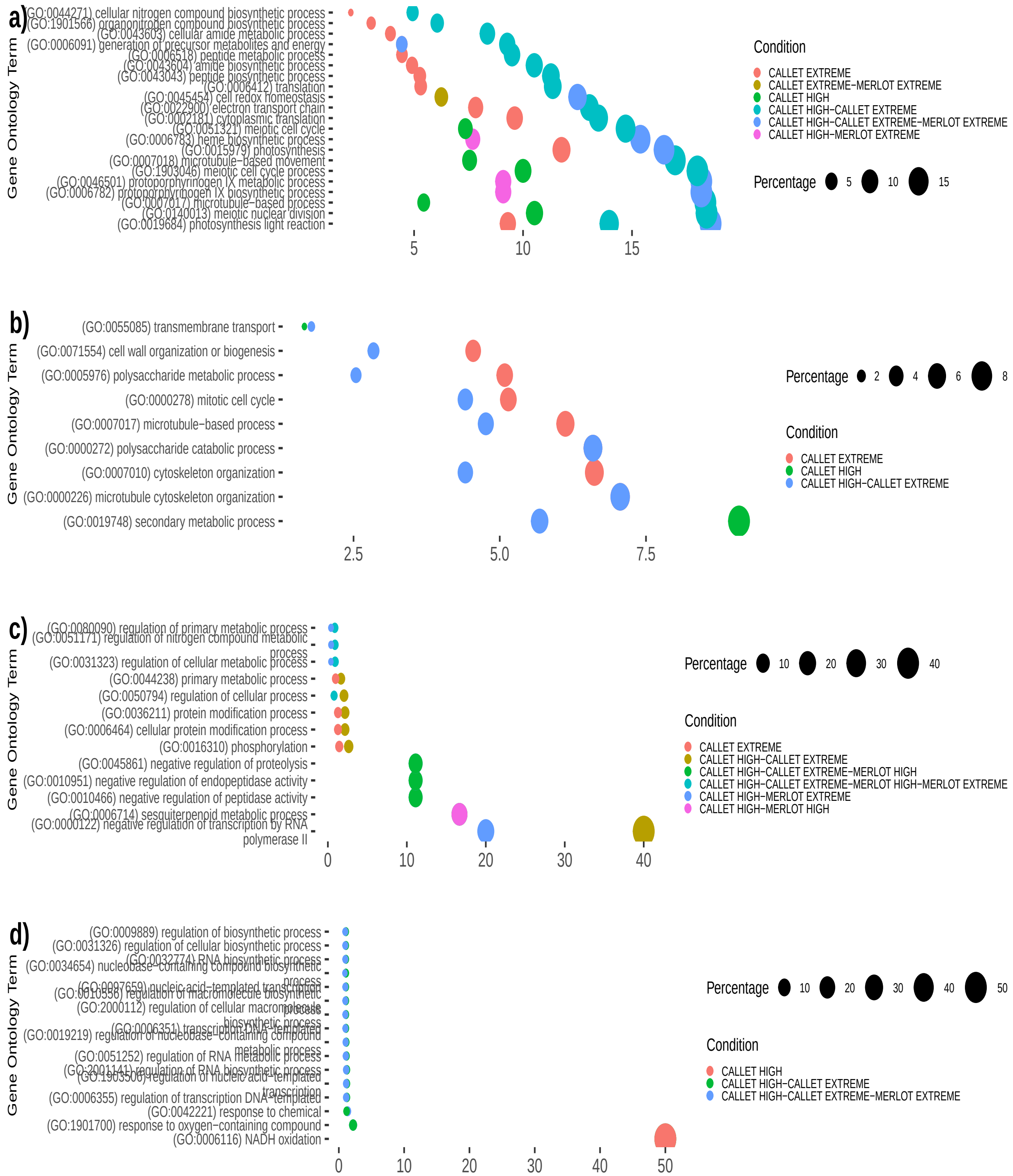

Figure 2: Heatmaps of the significant GOs based on P-value obtained in the Venn diagram (figure 5) for: a) upregulated genes in leaves; b) downregulated genes in leaves; in *Callet/110 Richter* and *Merlot/110 Richter*. The green scale represents the significancy of each GO in the total universe of grapevine GOs.

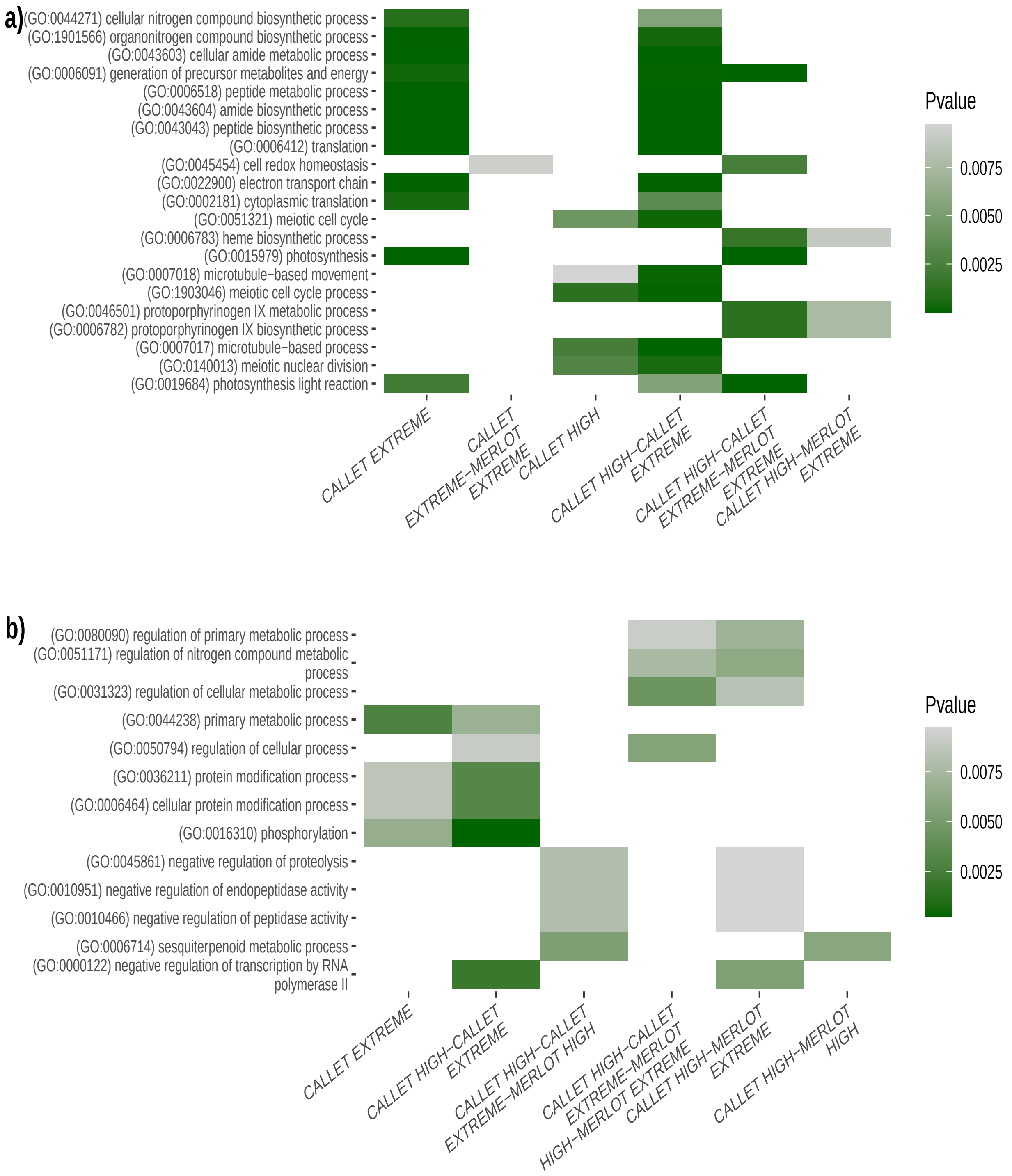

Figure 3: Heatmaps of the significant GOs based on P-value obtained in the Venn diagram (figure 5) for: a) upregulated genes in roots; and b) downregulated genes in roots; in *Callet/110 Richter* and *Merlot/110 Richter*. The green scale represents the significancy of each GO in the total universe of grapevine GOs.

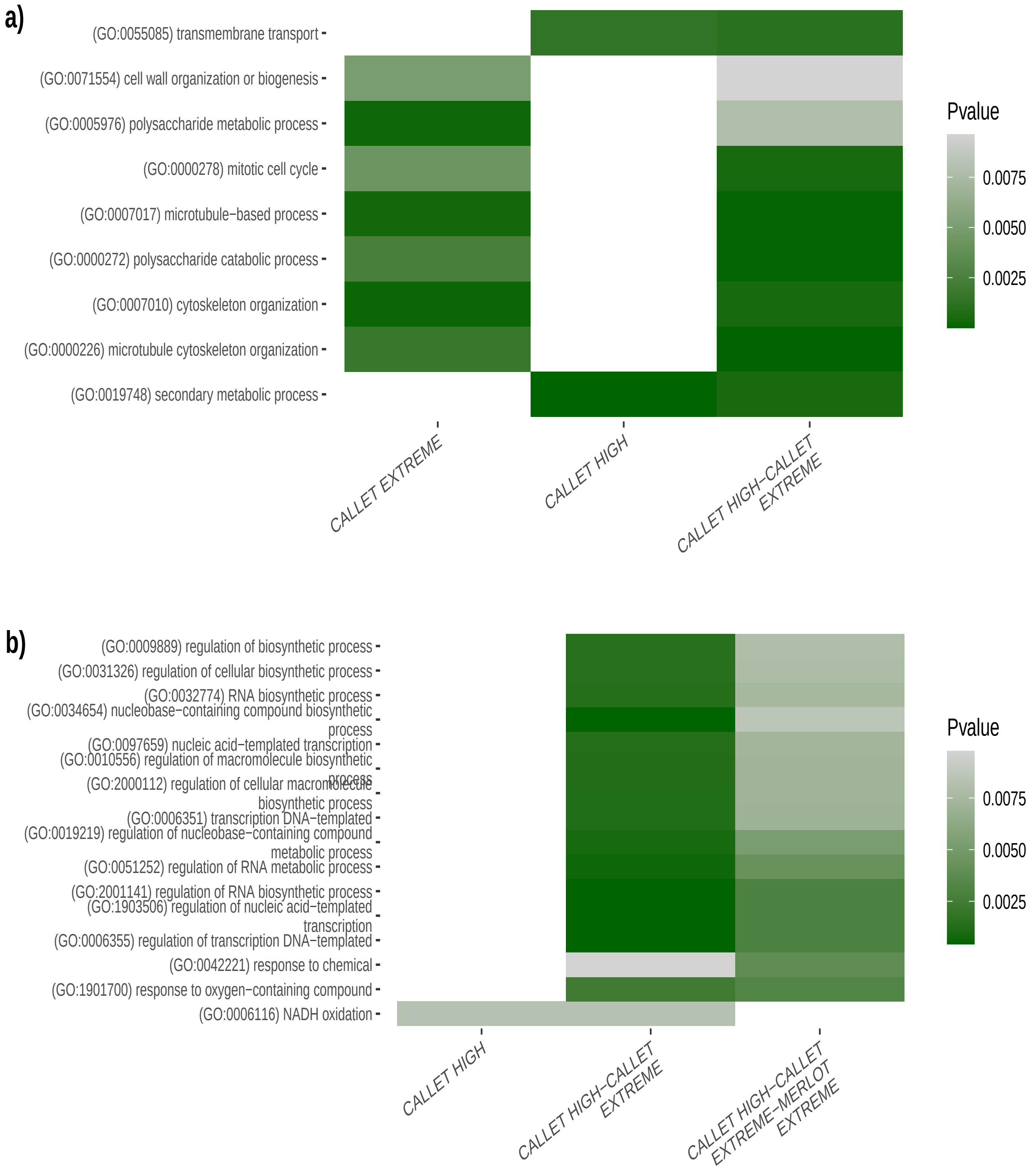

Figure 4: PCA scores for RNA-seq data on leaves of *Callet/110 Richter* and *Merlot/110 Richter* under different levels of water scarcity in 2020 and 2022. a) *Callet* Control in red and High in blue; b) *Callet* Control in red and Extreme in blue; c) *Callet* Control in red, High in blue and Extreme in green; d) *Merlot* Control in red and High in blue; e) *Merlot* Control in red and Extreme in blue; and f) *Merlot* Control in red, High in blue and Extreme in green.

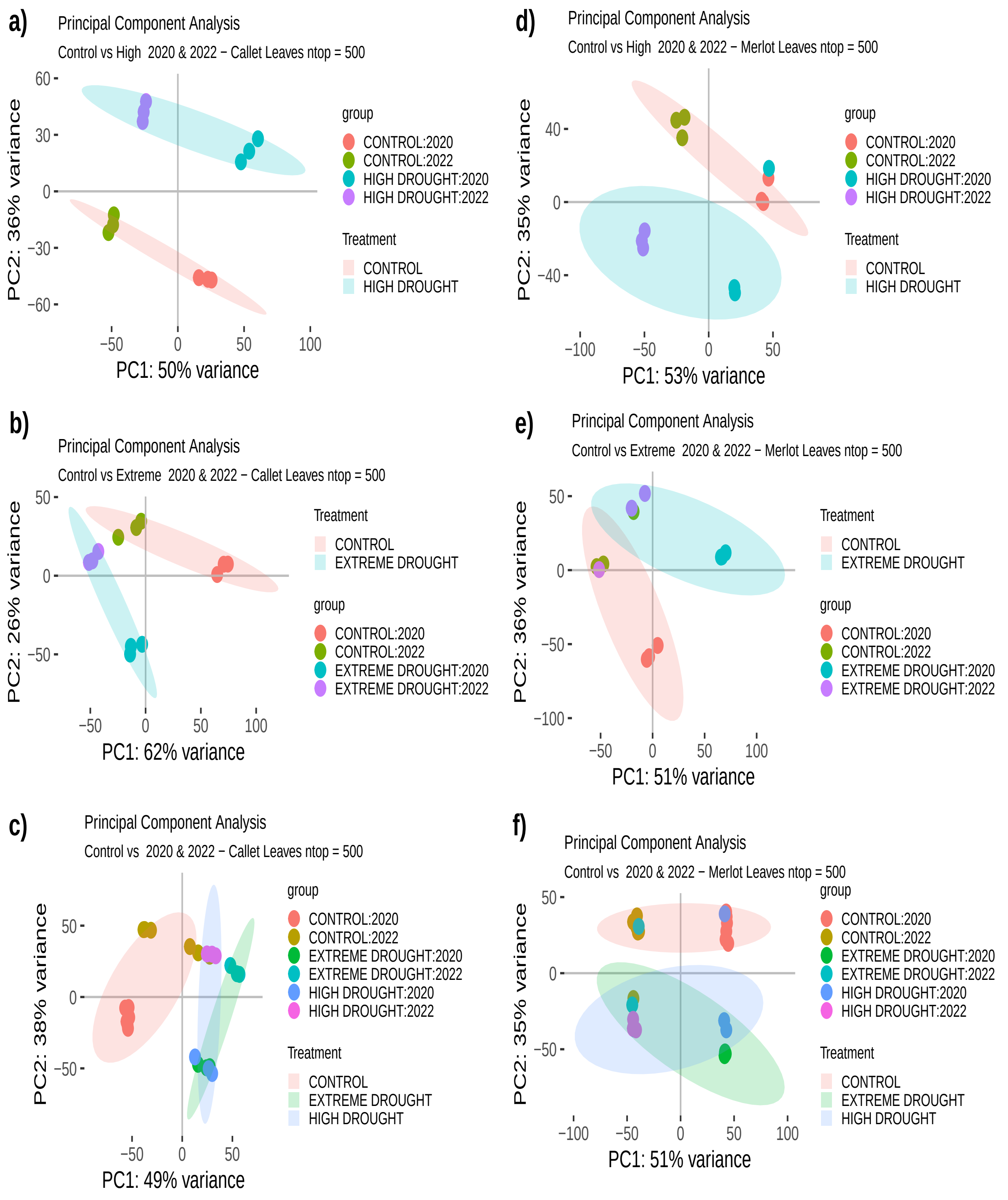

Figure 5: PCA scores for RNA-seq data on roots of *Callet/110 Richter* and *Merlot/110 Richter* under different levels of water scarcity in 2020 and 2022. a) *Callet* Control in red and High in blue; b) *Callet* Control in red and Extreme in blue; c) *Callet* control in red, High in blue and Extreme in green; d) *Merlot* Control in red and High in blue; e) *Merlot* Control in red and Extreme in blue; and f) *Merlot* Control in red, High in blue and Extreme in green.

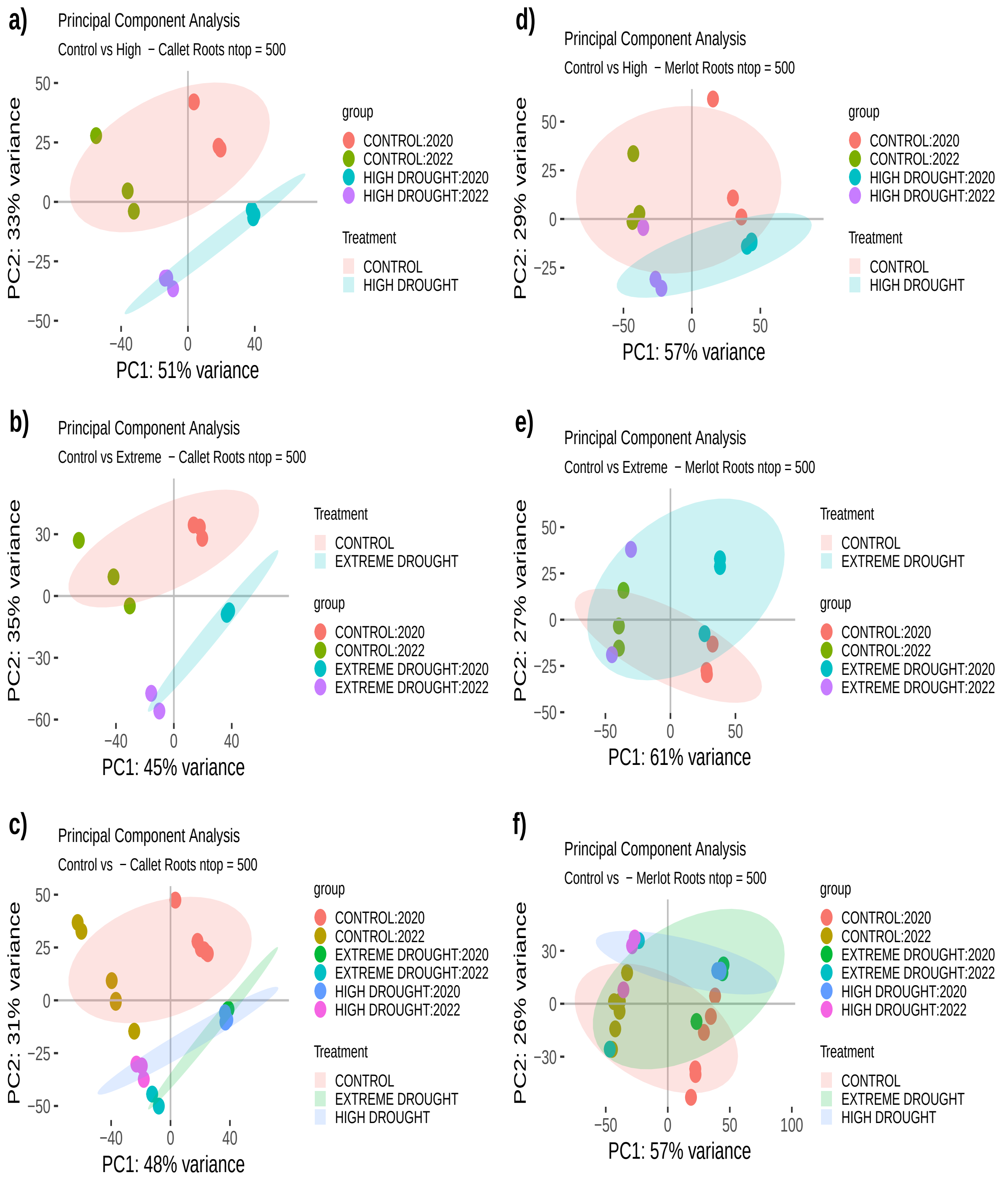

Figure 6: Representation of clusters of co-expressed genes detected in *k-means* analysis for RNA-seq results on *Callet/110 Richter* leaves for Control, High and Extreme water levels of water scarcity. For each cluster, it appears the cluster ID as a number and the number of genes corresponding to it. The black line represents the average of the normalized counts, and the grey shadow represents the dispersion of the counts around the average value for each condition.

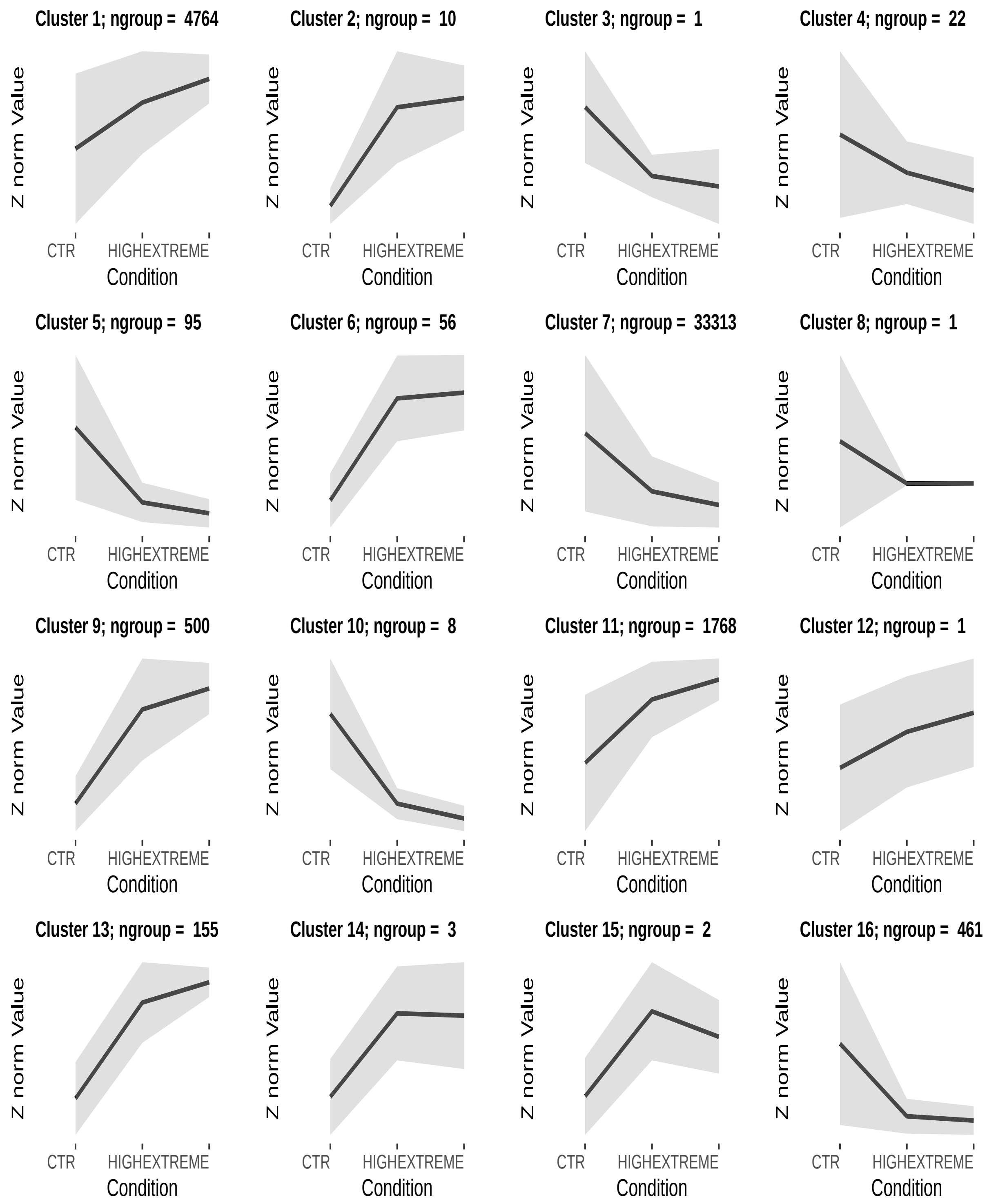

Figure 7: Representation of clusters of co-expressed genes detected in *k-means* analysis for RNA-seq results on *Merlot/110 Richter* leaves for Control, High and Extreme levels of water scarcity. For each cluster, it appears the cluster ID as a number and the number of genes corresponding to it. The black line represents the average of the normalized counts, and the grey shadow represents the dispersion of the counts around the average value for each condition.

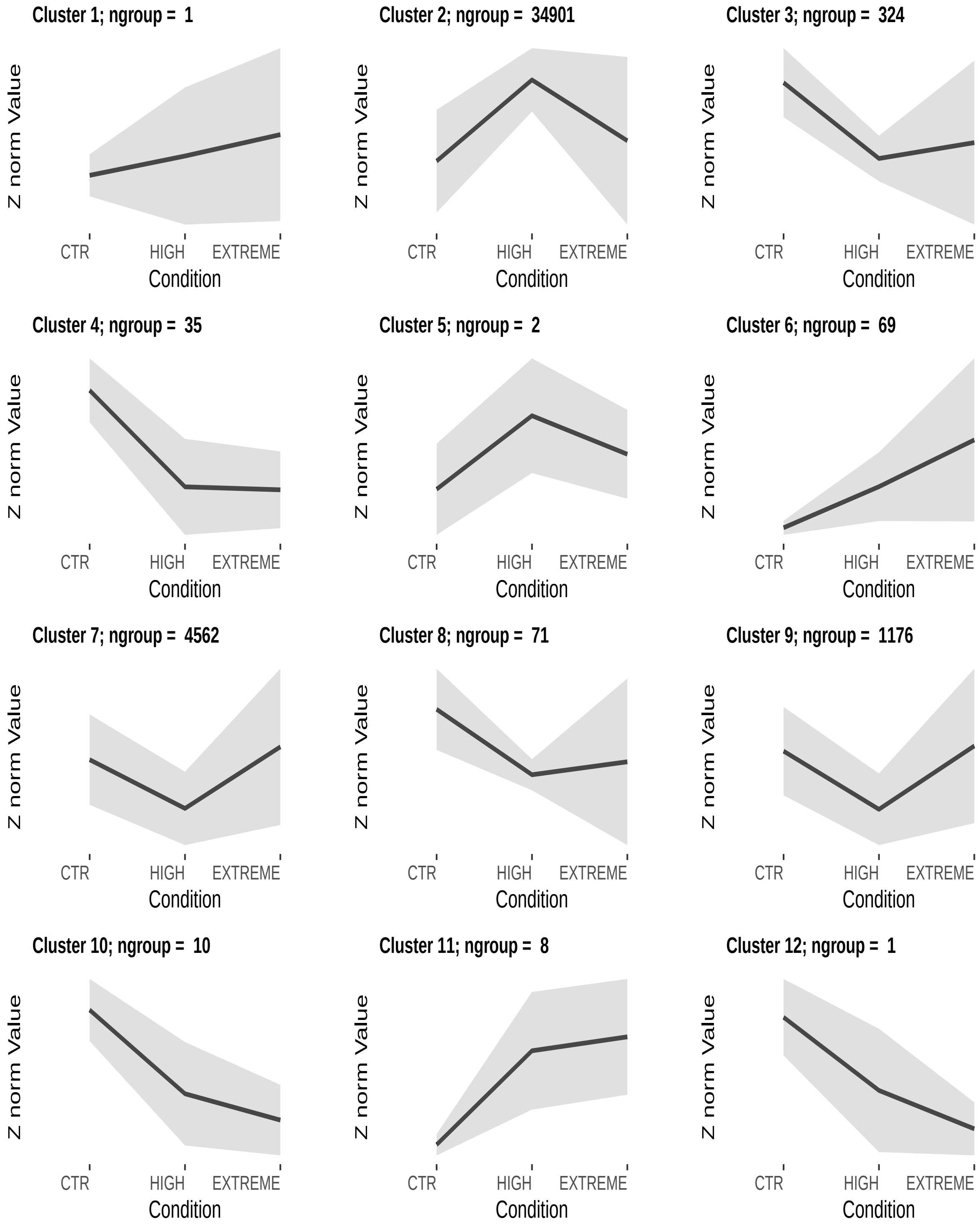

Figure 8: Representation of clusters of co-expressed genes detected in *k-means* analysis for RNA-seq results on *Callet/110 Richter* roots for Control, High and Extreme levels of water scarcity. For each cluster, it appears the cluster ID as a number and the number of genes corresponding to it. The black line represents the average of the normalized counts, and the grey shadow represents the dispersion of the counts around the average value for each condition.

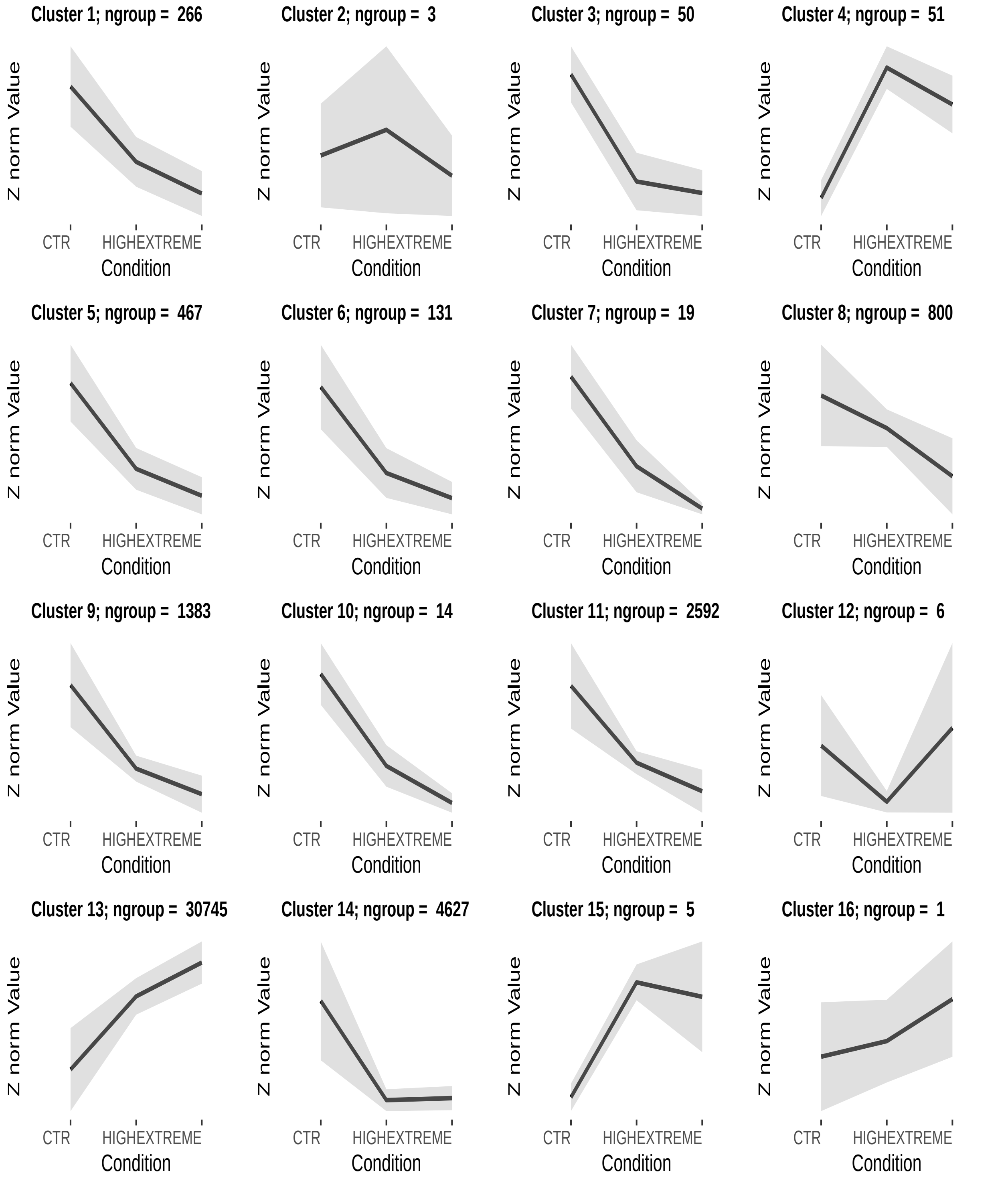

Figure 9: Representation of clusters of co-expressed genes detected in *k-means* analysis for RNA-seq results on *Merlot/110 Richter* roots for Control, High and Extreme levels of water scarcity. For each cluster, it appears the cluster ID as a number and the number of genes corresponding to it. The black line represents the average of the normalized counts, and the grey shadow represents the dispersion of the counts around the average value for each condition.

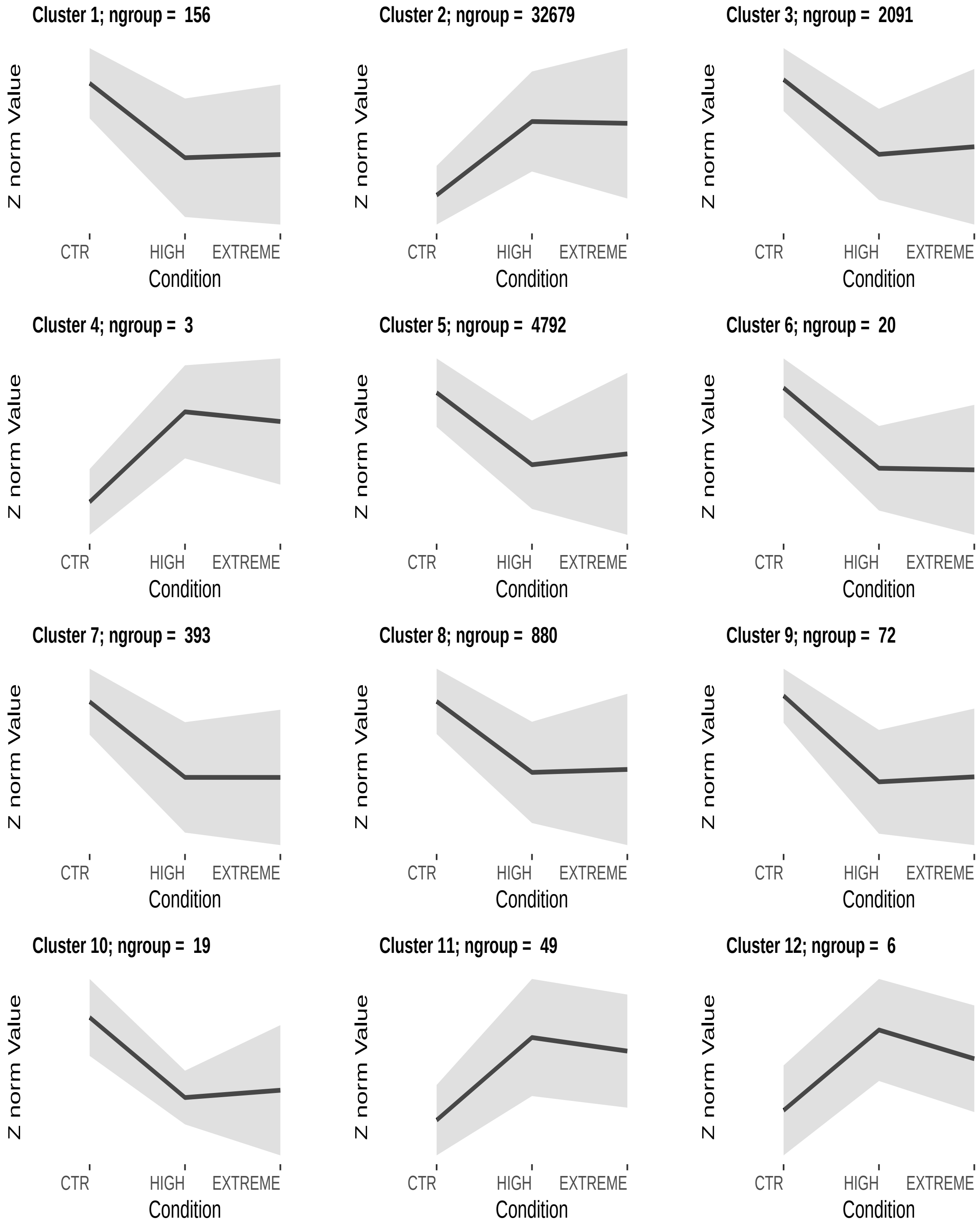

Supplement: Web_Material_uhae291 [file web_material_uhae291.zip › Supplementary_Material_2.pdf]
