# Supplementary material for: The scion-driven transcriptomic changes guide the resilience of grafted near-isohydric grapevines under water deficit
Source: Hortic Res. 2024 Oct 23;12(2):uhae291. doi: 10.1093/hr/uhae291 (PMC11789524; doi:10.1093/hr/uhae291)

Supplementary Material 3: The sensitivity to ABA in grapevine scion guides the coordination with rootstock under drought conditions.

Rodriguez-Izquierdo et al., 2024

Spain, 2024

Figure 1: Venn diagram for DEG genes on leaves of *Callet/110 Richter* (in red) and *Merlot/110 Richter* (in blue) under a) Mild, b) High and c) Extreme drought stages in 2020 and 2022.

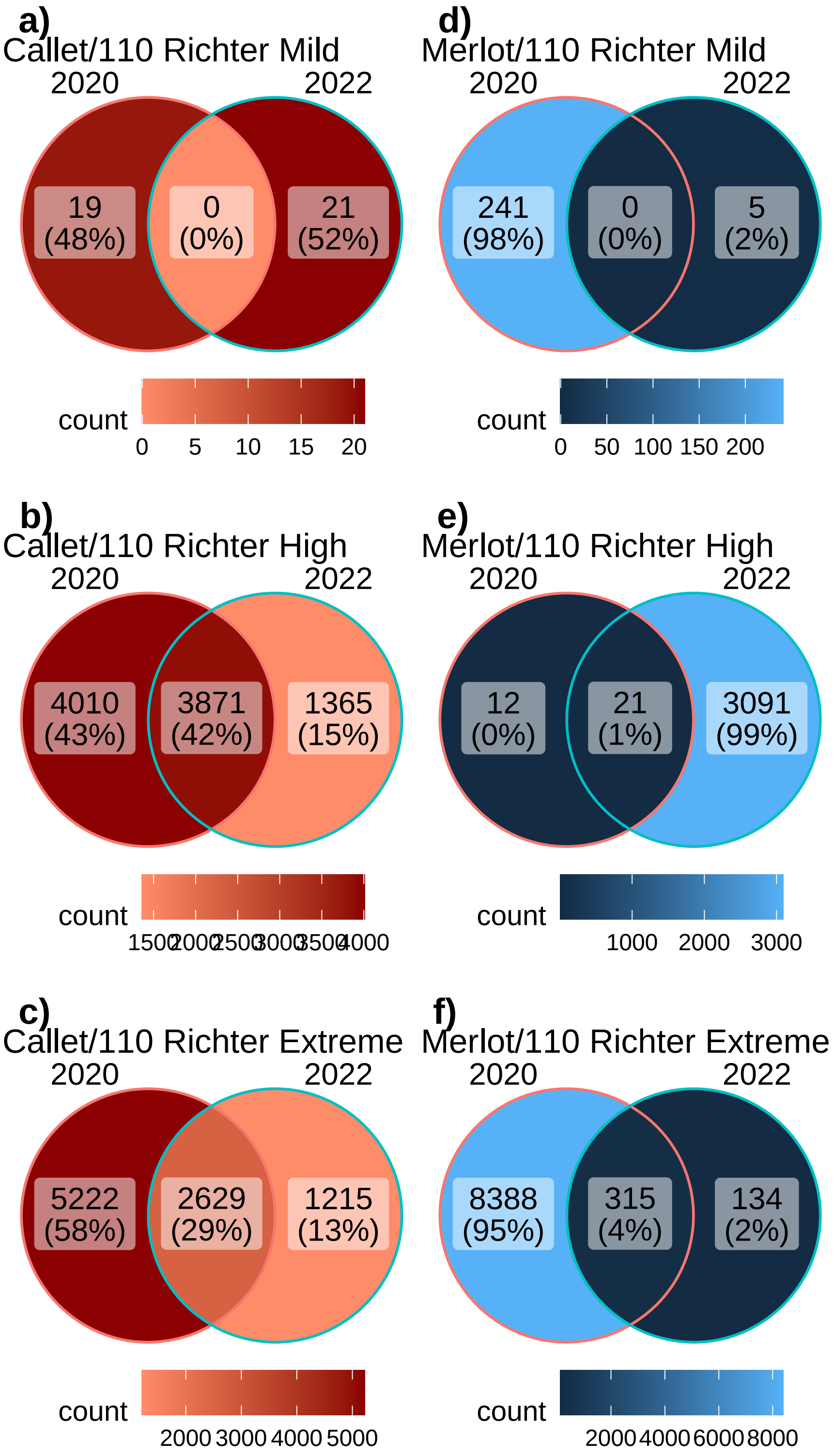

Figure 2: Venn diagram for DEG genes on roots of *Callet/110 Richter* (in red) and *Merlot/110 Richter* (in blue) under a) Mild, b) High and c) Extreme drought stages in 2020 and 2022.

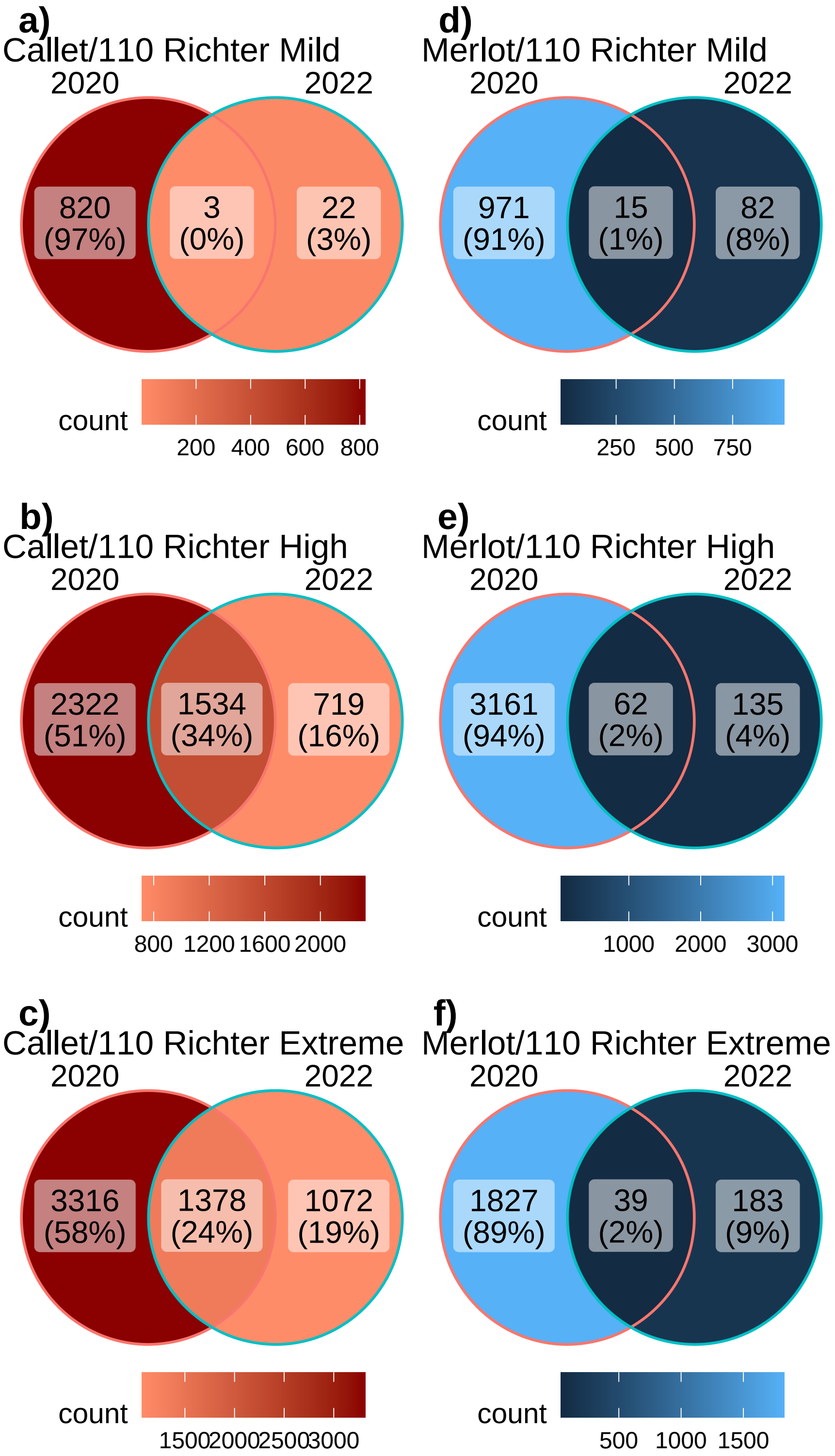

Supplement: Web_Material_uhae291 [file web_material_uhae291.zip › Supplementary_Material_3.pdf]
